# Antioxidant Molecules Isolated from Edible Prostrate Knotweed: Rational Derivatization to Produce More Potent Molecules

**DOI:** 10.1155/2022/3127480

**Published:** 2022-02-27

**Authors:** Mater H. Mahnashi, Bandar A. Alyami, Yahya S. Alqahtani, Ali O. Alqarni, Muhammad Saeed Jan, Fida Hussain, Rehman Zafar, Umer Rashid, Muhammad Abbas, Muhammad Tariq, Abdul Sadiq

**Affiliations:** ^1^Department of Pharmaceutical Chemistry, College of Pharmacy, Najran University, Najran, Saudi Arabia; ^2^Department of Pharmacy, University of Swabi, KP, Pakistan; ^3^Department of Pharmaceutical Chemistry, Faculty of Pharmaceutical Sciences, Riphah International University, Islamabad 44000, Pakistan; ^4^Department of Chemistry, COMSATS University Islamabad, Abbottabad Campus, 22060 Abbottabad, Pakistan; ^5^Department of Pharmacy, Abdul Wali Khan University Mardan, 23200 Mardan, KP, Pakistan; ^6^Department PCB, Rokhan University, Jalalabad, Nangrahar, Afghanistan; ^7^Department of Pharmacy, Faculty of Biological Sciences, University of Malakand, Chakdara, 18000 Dir (L), KP, Pakistan

## Abstract

Prostrate knotweed also called *Polygonum aviculare* is an important edible plant. The polygonum is majorly known for the phenolics and antioxidants. The antioxidants combat the excessive free radicals within the body. The excessive free radicals are implicated in various other diseases like diabetes, Alzheimer's, and inflammation. This study was aimed at exploring the antioxidant bioactives and their derivatizations to produce new molecules with advanced pharmacological features. We have isolated six compounds (**1–6**) from *Polygonum aviculare*. Furthermore, rational-based chemical derivatives for compound **5** have been formed for the management of diabetes, Alzheimer's, and inflammation. In preliminary antioxidant studies, all the isolated compounds (**1–6**) showed potential results against DPPH and ABTS free radicals. Based on the IC_50_ and chemical nature of the compounds, compound **5** was subjected to derivatization. Keeping the phenolic part of compound **5** unaffected, hydroxy succinimide (**5A**) and thiazolidinedione (**5B**) were synthesized. The compound **5A** was found to be a potent inhibitor of AChE, BChE, COX-1, COX-2, 5-LOX, and DPPH giving IC_50_ values of 10.60, 15.10, 13.91, 1.08, 0.71, and 1.05 *μ*M, respectively. The COX-2 selectivity of compound **5A** was found at 12.9. The compound **5B** was found to be a potent multitarget antidiabetic agent giving IC_50_ values of 15.34, 21.83, 53.28, and 1.94 *μ*M against *α*-glucosidase, *α*-amylase, protein tyrosine phosphatase 1B, and DPPH. Docking studies were performed to manipulate the binding interactions. The docking pose of all the tested compounds was found to have increased binding affinity against all tested targets that supported the *in vitro* results. Our results showed that *Polygonum aviculare* is a rich source of antioxidant compounds. The two new derivatives have enhanced pharmacological features to treat diabetes, inflammation, and Alzheimer's disease.

## 1. Introduction

Natural edible plants have a vital role in human health with no side effects [1]. The prostrate knotweed is an edible plant and can be directly used as healthy food especially in diarrhea [2]. The Knotgrass Mead is one of the beverage brands which is made up of 100% pure natural ingredient. This brand contains knotgrass and pure fermented honey. The knotgrass (*Polygonum aviculare*) extract is previously reported to have strong antioxidant properties [3]. The human being from ancient era is using medicinal plants for various ailments. Even in the recent time, the medicinal plant research is in routine practice [4]. The medicinal plants can be used for any disease condition based on folkloric, ethnomedicinal, and scientific backgrounds [5]. One of the strongest rationale for using a medicinal plant for a specific pharmacological target is its ethnomedicinal background [6]. Sometimes, a new plant can be explored for research studies. In the case of a new plant, the ethnomedicinal background of the plant is considered [7]. Herbal medicine or medicinal plant is a great source for the management of vital diseases like Alzheimer's disease, diabetes, cancer, infectious diseases, analgesia, and inflammation [8].

The bioactive compounds obtained from natural sources are considered relatively safer compared to the synthetic drug molecules [9]. A medicinal plant is a great source of bioactive compounds like steroids, phenolic, flavonoids, glycosides, and other bioactive phytoconstituents [10]. One of the easy approaches to confirm the presence of groups of compounds is the preliminary phytochemical testing [11]. To get a better and authentic idea about the phytocomponents, the GC-MS or LC-MS is a reliable approach which provides information about exact structure match [12]. In the recent years, several bioactive compounds have been isolated as potential pharmacological moieties from different plants [13]. Different furan analogs have been isolated as potential anticancer compounds from *Polygonum barbatum* [6]. Similarly, steroidal phytocomponent has also been isolated from *Polygonum hydropiper* as potential bioactive compounds [14]. The literature also confirmed the isolation of flavonoid-type compounds from *Polygonum aviculare* [15].

Alzheimer's disease is a challenging neurological disorder of the elder people. This disease causes an imbalance in life activities like cognitive dysfunction, behavioral and memory loss [16]. The two major neuropathologic points are beta-amyloid plaques (outside the cell) and neurofibrillary masses (inside the cell) [17]. The amyloid plaques are formed by breaking the amyloid precursor protein [18]. On the other side, neurofibrillary masses consist of tau protein responsible majorly for microtubule's stability [19]. In Alzheimer's patients, acetylcholine within the synaptic region splits to produce abundance of acetyl and choline. Splitting of the natural neurotransmitter acetylcholine is speedup by the biocatalysts cholinesterases [20]. The inhibitions of these biocatalysts (cholinesterases) are one of the major biochemical targets for the management of Alzheimer's disease [21].

The cell or tissue damage is autoprotected from viral, microbial, chemical, or physical injury by the process of inflammation [22]. In inflammation, blood vessel dilation occurs which increases the intracellular space [23]. The arachidonic acid pathway plays a vital role in the mechanism of inflammation. The cyclooxygenase and lipoxygenase metabolize arachidonic acid into prostaglandins and leukotrienes [24]. The inhibitions of cyclo- and lipoxygenase reduce the prostaglandin and leukotriene levels both of which play a visible role in the inflammatory process [25]. Thus, drug molecules capable of inhibiting these enzymatic pathways will have anti-inflammatory properties.

The hyperglycemia (diabetes mellitus) is a metabolic disease and is majorly caused by the impairment or dysfunction of beta cell which is responsible for insulin [26]. The long-term onset of this metabolic disorder affects the vital organ functions of the body [27]. High level of blood glucose level, slow wound healing, frequent urination, weight loss, blurred vision, and fatigue are among the most common symptoms of diabetes mellitus [28]. The major biochemical targets to reduce blood glucose level or improve insulin receptor functions are alpha glucosidase, alpha amylase, and protein tyrosine phosphatase 1B [29].

Antioxidants are majorly obtained from medicinal plants [30]. The antioxidants play a vital role in combating the human health issues by quenching the excessive free radicals within the body. These free radicals or reactive oxygen species within the body are implicated in the severity of various diseases like Alzheimer's disease, diabetes, analgesia, and inflammation [31–33]. In Alzheimer's, beta-amyloid plaques generate excessive free radicals and interrupt the mitochondrial function which ultimately causes neurodegeneration [34]. The excessive free radicals within the body cause lipid peroxidation and glycation of protein and also damage beta cells, thus implicating diabetes [35]. During the process of inflammation, the free radicals within the body damage the health cells also and thus increase the inflammation [36]. The immune system of the body can combat these free radicals [37]. However, this is practically not possible to combat excessive production of these free radicals. Therefore, the antioxidant can be used which synergistically suppresses these free radicals and hence reinstates the normal physiofunction. Among the plant phytochemicals, the phenolic and other alcoholic types of compounds are majorly responsible for antioxidant actions [38]. Based on the importance of phenolic compounds as potential antioxidant, this research was carried out to isolate such compounds from *Polygonum aviculare* as potential antioxidants.

## 2. Materials and Methods

### 2.1. General Experiment and Chemicals

The nuclear magnetic resonance (NMR) analyses were performed by using the Jeol-ECX400 instrument operating at 400 MHz for proton NMR while 100 MHz for carbon NMR. The chemical shifts of the splitting patterns were compared to the tetramethylsilane (TMS standard at 0). The TMS (0.03%) was present within the chloroform-d or DMSO-d_6_. The chemical reactions were performed in a small 2 mL reaction vessel. The precoated silica gel aluminum plates were used for the routine thin layer chromatography analysis. The chemicals and standard drugs used in this research were purchased from the local supplier of Sigma-Aldrich. All the solvents used were of analytical grades.

### 2.2. Plant Material and Extraction

The plant *Polygonum aviculare* was collected from District Mardan, KP, Pakistan, in the month of June. The plant was identified and confirmed by two botanists Dr. Nasrullah and Dr. Gul Rahim, Department of Botany, University of Malakand, Pakistan. The plant sample was registered in the herbarium of the University of Malakand with voucher number H.UOM. BG.556. The aerial parts of the plant were shade dried for three weeks. After shade drying, the plant materials were cut into small pieces (powdered). The powdered plant materials were added in 10 liters of ethanol (80%) for maceration of the plant materials. The plant materials (in ethanol) were examined on daily basis and shacked occasionally for better maceration. After fifteen days, the materials were filtered, and the residue was washed three times with small amount of ethanol to wash it out properly. The filtrate was concentrated at 40°C with the help of a rotary evaporator [5].

### 2.3. Isolations and Characterizations of Compounds

Initially, the crude plant material was loaded onto a prepacked silica gel gravity column. The process of elution was started with nonpolar solvent, i.e., *n*-hexane. A gradual increase in the solvent polarity was performed by addition of a polar modifier ethyl acetate. At the end of the column chromatography, semipurified two portions were obtained. Each of the semipurified portion was loaded onto a relatively small dimension prepacked silica gel gravity column. The column was eluted with combination of n-hexane, dichloromethane, and methanol. At the end of chromatography, six compounds (**1–6**) were purified and were observed as single spots on the TLC. The compounds were characterized by ^1^H NMR, ^13^C NMR, and mass analyses [6].

### 2.4. Chemistry of Isolated Compounds

**Furan-2-ylmethanol (1)**: MS data: 98 (molecular ion peak, 78%), 87 (14%), 81 (55%), 78 (76%), 69 (42%), 60 (100%), 55 (41%), 50 (22%), and 41 (43%) as shown in Figure S1. ^1^H NMR (CDCl_3_, 400 MHz) *δ*: 7.38 (d, *J* = 7.74 Hz, 1H), 6.35 (dd, *J* = 4.98, 9.01 Hz, 1H), 6.26 (d, *J* = 7.89 Hz, 1H), 4.53 (s, 2H), and 2.69 (s, 1H) as shown in Figure S2. ^13^C NMR (CDCl_3_, 100 MHz) *δ*: 155.62, 143.04, 110.79, 108.31, and 55.50 as shown in Figure S3.

**2,4-Dihydroxy-2,5-dimethylfuran-3(2H)-one (2)**: MS data: 144 (molecular ion peak, 48%), 101 (100%), 73 (80%), 55 (76%), and 43 (98%) as shown in Figure S4. ^1^H NMR (CDCl_3_, 400 MHz) *δ*: 6.25 (s, 1H), 4.54 (s, 1H), 2.25 (s, 3H), and 1.77 (s, 3H) as shown in Figure S5. ^13^C NMR (CDCl_3_, 100 MHz) *δ*: 198.70, 149.02, 128.99, 101.71, 24.63, and 21.14 as shown in Figure S6.

**4-Vinylphenol (3)**: MS data: 120 (molecular ion peak, 100%), 98 (2%), 91 (58%), 85 (8%), 75 (4%), 65 (20%), and 46 (4%) as shown in Figure S7. ^1^H NMR (CDCl_3_, 400 MHz) *δ*: 7.31 (d, *J* = 8.8 Hz, 2 H), 6.84 (d, *J* = 8.8 Hz, 2H, 6.65-6.57 (m, 1H), 5.65 (dd, *J* = 10.53, 9.23 Hz, 1H), 5.28 (s, 1H), and 5.15 (dd, *J* = 4.11, 10.63 Hz, 1H) as shown in Figure S8. ^13^C NMR (CDCl_3_, 100 MHz) *δ*: 155.06, 141.06, 131.57, 129.81, 121.69, and 116.45 as shown in Figure S9.

**2-Methoxy-3-methylbenzene-1,4-diol (4)**: MS data: 154 (molecular ion peak, 100%), 146 (21%), 139 (58%), 111 (36%), 103 (83%), 93 (31%), 85 (62%), 74 (20%), 61 (44%), 51 (18%), and 43 (75%) as shown in Figure S10. ^1^H NMR (CDCl_3_, 400 MHz) *δ*: 6.62 (d, *J* = 7.95 Hz, 1H), 6.33 (d, *J* = 7.94 Hz, 1H), 6.26 (s, 1H), 6.15 (s, 1H), 3.88 (s, 3H), and 2.24 (s, 1H) as shown in Figure S11. ^13^C NMR (CDCl_3_, 100 MHz) *δ*: 151.56, 144.92, 124.57, 122.44, 117.83, 114.33, 55.36, and 13.15 as shown in Figure S12.

**4-Hydroxy-3-methoxybenzaldehyde (5)**: MS data: 151 (molecular ion peak, 100%), 136 (23%), 121 (25%), 109 (38%), 81 (37%), 69 (50%), and 57 (36%) as shown in Figure S13. ^1^H NMR (CDCl_3_, 400 MHz) *δ*: 9.82 (s, 1H), 7.44-7.41 (m, 2H), 7.04 (d, *J* = 8.51 Hz, 1H), 6.27 (s, 1H), and 3.96 (s, 3H) as shown in Figure S14. ^13^C NMR (CDCl_3_, 100 MHz) *δ*: 191.02, 151.76, 147.22, 129.95, 127.64, 114.45, 108.82, and 56.20 as shown in Figure S15.

**1-(4-Hydroxy-3-methoxyphenyl)propan-2-one (6)**: MS data: 180 (molecular ion peak, 22%), 159 (17%), 137 (100%), 122 (18%), 94 (15%), 61 (8%), and 43 (17%) as shown in Figure S16. ^1^H NMR (CDCl_3_, 400 MHz) *δ*: 7.07 (d, *J* = 8.05 Hz, 1H), 6.85 (d, *J* = 8.04 Hz, 1H), 6.56 (s, 1H), 6.24 (s, 1H), 3.95 (s, 3H), 3.53 (s, 2H), and 2.08 (s, 3H) as shown in Figure S17. ^13^C NMR (CDCl_3_, 100 MHz) *δ*: 201.19, 145.51, 143.24, 116.82, 116.67, 115.83, 57.24, 52.65, and 41.08 as shown in Figure S18.

### 2.5. Chemical Derivatization of Compound **5**

#### 2.5.1. Synthesis of Compound **5A**

To a solution of 3-methoxy-4-hydroxybenzaldehyde (compound **5** in ethanol), 1-(4-hydroxyphenyl)pyrrolidine-2,5-dione and piperidine in small amount were added. The reaction was continued for 24 hours. At the end of reaction, the precipitates appeared which were crystallized with the help of dimethylformamide and ethanol. The isolated compound **5A** was confirmed with ^1^H NMR and ^13^C NMR analyses [39].

**(E)-3-(4-Hydroxy-3-methoxybenzylidene)-1-(4-hydroxyphenyl)pyrrolidine-2,5-dione (5A)**: ^1^H NMR (DMSO-d6, 400 MHz) *δ*: 7.82 (s, 1H), 7.49-7.44 (m, 2H), 7.11-7.07 (m, 1H), 6.96 (d, *J* = 7.91 Hz, 1H), 6.82 (d, *J* = 7.53 Hz, 1H), 6.50 (s, 1H), 6.25 (s, 1H), 3.91 (s, 3H), and 3.63-3.48 (m, 2H) as shown in Figure S19. ^13^C NMR (DMSO-d6, 100 MHz) *δ*: 177.88, 174.75, 152.64, 149.41, 146.28, 141.39, 133.83, 130.92, 130.67, 130.30, 125.01, 123.52, 122.30, 121.89, 119.55, 118.17, 54.77, and 37.72 as shown in Figure S20.

#### 2.5.2. Synthesis of Compound **5B**

The previously reported procedure was followed for the synthesis of compound **5B**. To a solution of 3-methoxy-4-hydroxybenzaldehyde (compound **5** in ethanol), thiazolidine-2,4-dione and piperidine in small amount were added. The reaction was continued for 24 hours. At the end of reaction, the precipitates appeared which were crystallized with the help of dimethylformamide and ethanol. The isolated compound **5B** was confirmed with ^1^H NMR and ^13^C NMR analyses [40].

**(Z)-5-(4-Hydroxy-3-methoxybenzylidene)thiazolidine-2,4-dione (5B)**: ^1^H NMR (DMSO-d6, 400 MHz) *δ*: 12.92 (s, 1H), 7.73 (s, 1H), 7.38 (d, *J* = 2.47 Hz, 1H), 7.19 (d, *J* = 1.84, 3.74 Hz, 1H), 6.91 (d, *J* = 3.30 Hz, 1H), 6.20 (s, 1H), and 3.94 (s, 3H) as shown in Figure S21. ^13^C NMR (DMSO-d6, 100 MHz) *δ*: 177.61, 175.55, 153.07, 146.46, 142.23, 135.99, 119.31, 117.06, 114.21, 109.71, and 54.69 as shown in Figure S22.

### 2.6. In Vitro Pharmacology

#### 2.6.1. ABTS Assay

The ABTS free radical scavenging antioxidant activity of the compounds was performed by using the previously reported methods. In this activity, the compounds scavenge ABTS^+^ cations, and thus, a reduction in absorption is observed at 734 nm using a UV-visible spectrophotometer. Solutions of 7 mM ABTS and 2.45 mM of K_2_S_2_O_4_ were prepared and were mixed. The mixture was stored at dark for about 16 h to get ABTS cations which appeared dark in color. The solution of ABTS cation was diluted with phosphate buffer (0.01 M having pH of 7.4); the absorbance value (0.70) was adjusted at 734 nm. To confirm the ABTS radicals' scavenging capacity, 300 *μ*L solution of compound was mixed with 3.0 mL of ABTS. After one minute, the reduction in absorption was noted and was monitored for six minutes. The ascorbic acid was used as positive control in this activity. The experiments were repeated three times. The percent inhibitions and its IC_50_ values were measured as per the standard method [41].

#### 2.6.2. DPPH Assay

The 1,1-diphenyl-2-picrylhydrazyl free radicals of the compounds were performed by using the reported method [42]. Different concentrations of the compounds were mixed with DPPH methanolic solution of 0.004%. After half an hour, the absorption was determined with a double-beam UV-visible spectrophotometer at 517 nm. The same standard (ascorbic acid) was used as in the ABTS assay. The experiments were repeated three times. The percent inhibitions and IC_50_ values of the compounds were calculated with the standard formula.

#### 2.6.3. Anticholinesterase Assays on Compound **5A**

The acetyl and butyrylcholinesterase activities were performed on compound **5A** in comparison to the standard drug galantamine. The two enzymes break down their respective thiocholine iodide to produce the ion 5-thio-2-nitrobenzoate. The complex formed (yellow color anion) was confirmed with a spectrophotometer. Various dilutions of the compound **5A** were formed. The enzyme dilutions were prepared in phosphate buffer having pH of 8. Parallelly, the acetyl and butyrylthiocholine iodide solutions (0.05 mM) and DTNB were also prepared in H_2_O (distilled water of our laboratory) and were stored at 8°C for 15 min. Different dilutions of galantamine were also prepared in methanol. The enzyme, compound **5A**, and DTNB solutions 5, 205, and 5 *μ*L, respectively, were mixed and incubated for 15 min at 30°C. The absorbance was measured at 412 nm. The negative control served was all the solutions except compound **5A** while galantamine was the positive control. The experiments were performed three times. The percent inhibitions and their subsequent IC_50_ values were calculated as per the standard formula [43].

#### 2.6.4. Anticyclooxygenase Assays (COX-1/COX-2) on Compound **5A**

Based on the rationale of our designed study, the COX-1 and COX-2 inhibitory assays were performed on compound **5A** only. The COX-1 enzyme 10 *μ*L and COX-2 300 units/mL were activated on ice for 5 min. The activation solution was accompanied by a cofactor of 50 *μ*L containing glutathione, hematin, 1.0 mM, and TMPD in Tris-HCl buffer with pH of 8. The enzyme solutions 60 *μ*L and compound **5A** 20 *μ*L with various dilutions were kept at normal laboratory temperature for 5 min. The reaction was initiated when arachidonic acid 20 *μ*L was added. The reaction was incubated for 5 min, and absorbance was measured at 570 nm as per the reported procedure. The experiments were repeated three times. The percent inhibitions with its subsequent IC_50_ values were calculated as per the standard reported formula and were compared with the standard diclofenac [44].

#### 2.6.5. 5-Lipoxygenase Assay (5-LOX)

The 5-LOX activity was also performed on compound **5A** and was compared with the standard zileuton. The lipoxygenase enzyme 10,000 unit/*μ*L was used in the assay. The substrate used in this assay was linoleic acid. The compound **5A** was dissolved in phosphate buffer having pH of 6.3 followed by addition of the enzyme solution. The combined mixture was incubated at 25°C for 5 min. Afterwards, linoleic acid was properly mixed with it and was kept for 5 min. The absorbance was measured at 234 nm. The experiments were performed three times. The percent 5-LOX inhibitions and the IC_50_ values were calculated as per the standard formula [44].

#### 2.6.6. Alpha Glucosidase Assay

For the alpha glucosidase activity of compound **5B**, we followed the reported procedure. The solution of compound (250 microliter) and the standard drug acarbose were incubated with alpha glucosidase (1.0 U/mL). The glucosidase solution was prepared in phosphate buffer (100 mM having pH of 6.8). The incubation was performed at 37°C for 15 min. Then, a solution of 4-nitrophenylglucopyranose (5 mM, 250 microliter) in phosphate buffer (100 mM, pH 6.8) was prepared, and the mixture was incubated at 37°C for 15 min. Using the double-beam spectrophotometer, the absorbance of free 4-nitrophenol was checked at 405 nm. The percent inhibitions were calculated as per the reported formula. The IC_50_ values were calculated from the observed percent inhibition [40].

#### 2.6.7. Alpha Amylase Assay

In this research, the alpha amylase activity was also performed on compound **5B** only. Different concentrations of compound **5B** and acarbose were prepared, i.e., from 500 to 31.25 *μ*g/mL. The enzyme solutions were added to the test samples and were incubated at 37°C for 15 min. The starch as a substrate was added to the mixture. Afterwards, hydrochloric acid 1 M was added to it and the absorbance was measured at 580 nm. The experiments were repeated three times. The percent inhibitions and its subsequent IC_50_ were calculated as per the standard reported formula [45].

#### 2.6.8. Protein Tyrosine Phosphatase 1B Assay

The protein tyrosine phosphatase 1B assay was also performed on compound **5B**. In the PTP1B assay, 4-nitrophenylphosphate was used as substrate. The solution of dimethyl glutarate substrate in buffer of pH 7 was prepared to be used in the assay. The solution was prepared from the substrate used (1 mM), protein tyrosine phosphatase 1B (10 mM), and different concentrations of compound **5B**. The solution was incubated at 37°C for 15 min. The absorbance was measured at 405 nm. The experiments were performed three times. The percent inhibitions and their corresponding IC_50_ values were calculated as per the standard formula [46].

### 2.7. Docking Studies

#### 2.7.1. Docking Studies Using Autodock Vina 2.2.1

To identify the antioxidant potential of all synthesized six chemical moieties by the in silico method, docking studies were performed. These are the trustworthy tools to have good understanding of drug-receptor bindings. Induced fit model docking was carried out using Autodock Vina 2.2.1 interconnected with PyRx. Two-dimensional structures of all synthesized compounds were sketched as Mol file (∗.mol) using MarvinSketch 20.0 software. CHARMm force field was utilized to minimize the energy and add polar hydrogen in the BIOVIA Discovery Studio Visualizer while saving it as pdb format. Grid dimensions in Angstrom were set as center *X*: 62.2322, *Y*: 60.3700, and *Z*: 82.8575. The antioxidant activities including free radical scavenging and reducing capacities of synthesized compounds are associated with tyrosinase reduction. It could be related to their redox properties that make them hydrogen atom donors and allow them to scavenge free radicals. Three-dimensional structure of tyrosinase with cocrystallized inhibitor kojic acid was acquired from (http://www.rcsb.org/pdb) Protein Data Bank as PDB ID: 5I38 and saved in pdb format after removing the cocrystallized ligand and water molecules and adding polar hydrogen. Protein receptors and target molecule configurations were analyzed and selected depending upon their binding energies, mean root square deviation integrations. Different types of interactions including their bond strength were represented through Ligplot and PyMol software's latest versions [47, 48].

#### 2.7.2. Docking Studies Using MOE 2016

We performed molecular docking simulations using Molecular Operating Environment software (MOE 2016). Compound **5A** was docked into the active sites of AChE, BChE, COX-1, COX-2, and 5-LOX. The three-dimensional structures of all the enzymes were obtained from the Protein Data Bank with accession codes 1EVE, 4BDS, 1EQG, 1CX2, and 6N2W, respectively. Compound **5B** was docked into the active sites of *α*-glucosidase, *α*-amylase, and PTP-1B. The three-dimensional structure of *α*-glucosidase was constructed by using the homology model technique as per our previously reported procedure [40], while 3-D structures of *α*-amylase and PTP-1B were obtained from the Protein Data Bank with accession codes 4W93 and 1NNY, respectively. Before starting the docking on tested compounds, the protocol for the docking studies was validated by using the redock method. The measured RMSD values were within reasonable limits (<2.0 Å). Preparation of downloaded enzymes such as determination of binding sites, energy minimization, and 3-D protonation was performed by previously reported methods. Structures of the compounds were built using the builder option in MOE software. The built structures were then energy minimized using MMFF94X forcefield and 0.0001 gradient, and a database was built. The docking study was carried out using validated parameters (placement/refinement stage and scoring/rescoring functions). Interpretation of docking results was carried out by using MOE and Discovery Studio Visualizer [49].

## 3. Results and Discussion

### 3.1. Chemistry of Isolated Compounds

**Figure 1:**
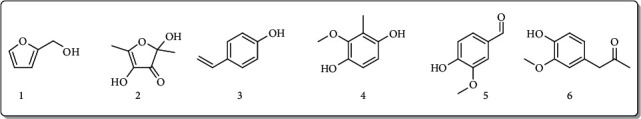


The structures of isolated compounds (**1–6**) from *Polygonum aviculare* are shown in Figure 1. All the compounds are polar in nature due to the presence of hydroxyl and other polar groups. Compounds **1** and **2** are furan-type derivatives. Similarly, compounds **3–6** can generally be classified as phenolic compounds to the presence of the Ph–OH group in their structures. However, all the isolated phenolic compounds are structurally different based on the attachment of other groups they contain. Two of these compounds (**5** and **6**) contain carbonyl groups, i.e., aldehyde and ketone, respectively. The analytical details of the isolated compounds are provided in the following.

### 3.2. Antioxidant Results of Compounds **1–6**

The antioxidant results of compounds **1–6** are summarized in Table 1. The two well-known free radicals' sources, i.e., ABTS and DPPH, were used to determine the antioxidant potentials of the compounds. Overall, all of our isolated compounds exhibited potent antioxidant results in both ABTS and DPPH assays. In the ABTS assay, compound **2** (2,4-dihydroxy-2,5-dimethylfuran-3(2H)-one) showed the highest antioxidant activity. Compound **2** exhibited percent inhibitions of 88.36, 83.34, 77.39, 71.47, and 66.44 at concentrations of 500, 250, 125, 62.5, and 31.25 *μ*g/mL, respectively, with calculated IC_50_ of 3.005 *μ*g/mL. In comparison, the standard ascorbic acid exhibited the IC_50_ value of 0.211 *μ*g/mL in the ABTS assay. Similarly, the next close potent compound in this assay was a dihydroxy-methoxy-toluene (**4**) type of phenolic compound. The observed IC_50_ of compound **4** was 4.63 *μ*g/mL.

In DPPH free radical scavenging assay, the majority of our compound showed relatively potent antioxidant activity. Similar to that of ABTS results, compound **2** was also the potent one among the tested compounds as shown in Table 1. The observed IC_50_ of compound **2** was 1.61 *μ*g/mL in comparison to the standard ascorbic acid which was 0.840 *μ*g/mL. The observed IC_50_ values for the remaining compounds in the DPPH assay were 8.19 (**1**), 10.27 (**3**), 3.19 (**4**), 2.12 (**5**), and 9.99 (**6**) *μ*g/mL. Based on the observed IC_50_ values, it is worth noting that compounds **4** and **5** are also potent antioxidant bioactive molecules.

**Scheme 1:**
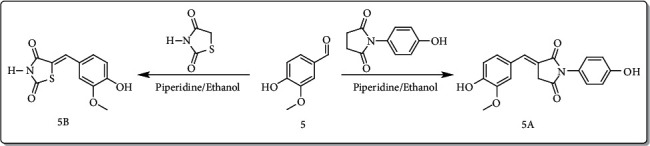


The free radicals are generally involved in the progression of various diseases like diabetes, Alzheimer's, and inflammation [50]. Thiazolidinedione is one of the most common classes of clinically practiced drugs for diabetes. Similarly, the succinimide is a known class of drugs for neuropharmacology and inflammation [44]. Various derivatives of succinimide-type compounds can be synthesized with different methods [51–54]. In our research, we noticed that compounds **2**, **4,** and **5** are potent antioxidant molecules. Based on these facts, we have structurally modified one of the antioxidant compounds (**5**) into a succinimide (**5A**) and a thiazolidinedione (**5B**) derivative as shown in Scheme 1. The purpose and rationale of compound **5B** are to introduce a molecule which has the capability to treat diabetes via multitarget approaches. Similarly, compound **5A** is designed in a way to treat Alzheimer's disease and inflammation.

### 3.3. Anticholinesterase, Anticyclo-and-Lipoxygenase Results of Compound **5A**

The free radicals are generally involved in the progression of various diseases like diabetes, Alzheimer's, and inflammation. In the antioxidant results of our compounds **1–6**, we noticed that compounds **2** and **5** have potent IC_50_ values specifically in the DPPH assay. Based on the chemical nature, we structurally derivatized compound **5** to a hydroxy succinimide. During this derivatization, the OH group of compound **5** was kept unaffected. The compound **5A** was designed rationally for its possible applications in neuroinflammation. We noticed that compound **5A** is more potent in antioxidant activity than the parent compound **5**. The observed IC_50_ value against DPPH free radicals for compound **5A** is 1.05 *μ*g/mL as shown in Table 2. Similarly, compound **5A** gave IC_50_ values of 10.60 and 15.10 *μ*g/mL against AChE and BChE, respectively. In comparison, the standard galantamine IC_50_ values were 4.0 and 15.0 *μ*g/mL against AChE and BChE, respectively. The calculated selectivity index was 1.42. The compound **5A** was also found to be a potent inhibitor of COX-1 and COX-2 enzymes giving IC_50_ values of 13.91 and 1.08 *μ*g/mL, respectively. The COX-2 selectivity index for our compound was found to be 12.9. This value shows that our compound **5A** is a selective COX-2 inhibitor having the advantage of low gastric toxicity associated with nonselective inhibitors. Moreover, we also find out the potency of compound **5A** against the 5-lipoxygenase enzyme. The observed IC_50_ value was 0.71 *μ*g/mL in comparison to the standard zileuton (5.29 *μ*g/mL). It shows that our compound is a relatively potent inhibitor of 5-lipoxygenase.

### 3.4. In Vitro Antidiabetic Results of Compound **5B**

Obviously, the free radicals are also implicated in diabetes [55]. So, based on this statement, we also rationally modified compound **5** to a thiazolidinedione derivative **5B**. Initially, we found that the derivatized compound **5B** has an IC_50_ value of 1.94 *μ*g/mL against DPPH free radicals. Further, this compound **5B** was designed for antidiabetic activities as the thiazolidinedione is a known class of antidiabetic. Compound **5B** exhibited IC_50_ values of 15.34, 21.83, and 53.28 *μ*g/mL in *α*-glucosidase, *α*-amylase, and protein tyrosine phosphatase 1B assays, respectively, as shown in Table 3.

### 3.5. Molecular Docking Studies

#### 3.5.1. Molecular Docking Studies of Compounds **1-6** toward Antioxidant Target

To understand the free radical scavenging antioxidant ability of all six synthesized compounds, docking studies were carried out. Antioxidants are a fundamental tool to fight against oxidative stress and aid the pathologies related to free radicals during various internal and external stress conditions [56]. In comparison with natural antioxidants, synthesized compounds also have various applications to reduce stress oxidation. In this regard, all compounds (**1-6**) were analyzed to study their free radical-induced oxidative stress-relieving ability (Table 4) but compounds **2** and **4** were further elaborated due to excellent *in vitro* results.

**Figure 2:**
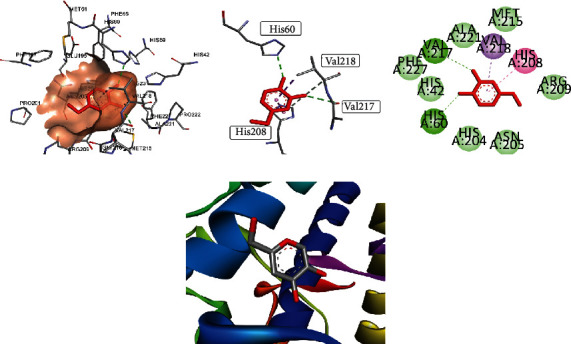


Docking studies elaborated that compound **2** gave binding energy of -6.987 Kcal/mol with scoring of -33.427 in the best binding posture among the top 10 poses, compared to cocrystallized inhibitor kojic acid as given in Figure 2. Docking studies were performed on tyrosinase with PDB ID: 5I38. The active site of tyrosinase has a knot of amino acid residue consisting of His A: 42, His A: 60, His A: 204, Asn A: 205, His A: 208, Arg A: 209, Met A: 215, Val A: 217, Val A: 218, Ala A: 221, and Phe A: 227. This widely dispersed copper-containing enzyme has a fundamental role in catalyzing the hydroxylation reaction of monophenol and diphenol oxidation reactions. Results of docking scoring supported the *in vitro* results.

**Figure 3:**
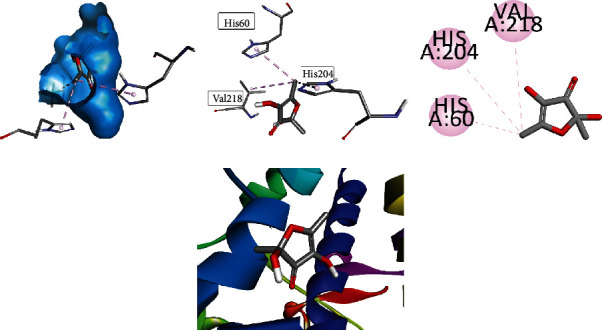


Compound **2** profound its interaction at the active site by binding with His A: 60, His A: 204, and Val A: 218. The most prominent interaction was alkyl interaction having bond length of 4.28 Å with His A: 60, bond length of 4.84 Å with His A: 204, and pi-alkyl bonding with five-membered ring having bond length of 4.87 Å with Val A: 218. All visualizations of compound **2** are shown in Figure 3.

**Figure 4:**
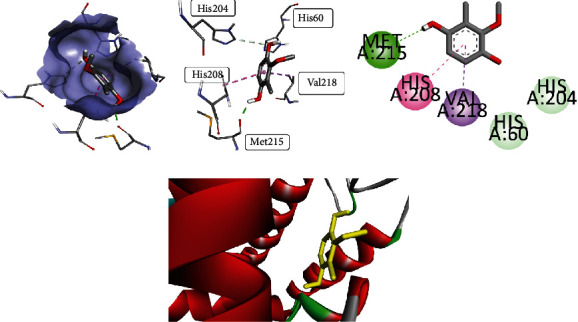


Compound **4** when analyzed for antioxidant potential synchronized with results obtained through in vitro activity. It gave the binding energy of -7.124 Kcal/mol with scoring of -39.834 in the best binding mode among the top 10 postures. The obtained conformations were analyzed to get the valuable information about the binding mode. The rational conformation of compound **4** indicated the favorable position of this compound inside the binding pocket. Compound **4** gave binding at the active site through conventional hydrogen bond having bond length of 1.99 Å, carbon0hydrogen bond having bond length of 3.50 Å–2.52 Å, and pi-sigma bonding with five-membered ring having bond length of 3.56 Å. Pi-pi stacked bonding was observed with His A: 208 with bond length of 4.24 Å. Compound **4** interaction visualizations are shown in Figure 4. It gave the best interaction with amino acid residue of binding pocket as His A: 60, His A: 204, His A: 208, Met A: 215, and Val A: 218 while Van der Waal forces were observed with His A: 42, His A: 205, and Phe A: 227.

#### 3.5.2. Docking Studies of Compounds **5A** and **5B** against Antioxidant Target Using Autodock Vina Synthesized Compounds **5A** and **5B**

**Figure 5:**
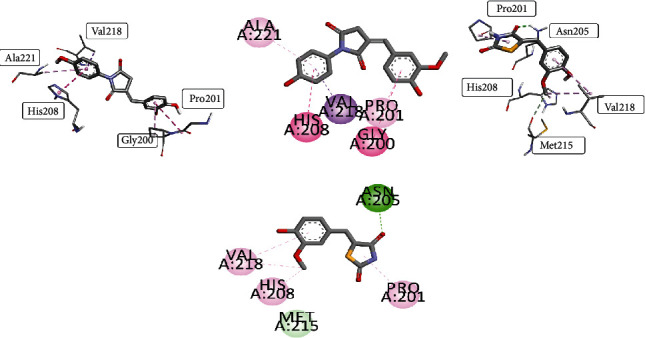


For manipulation of antioxidant potential of vanillin derivatives of compound **5**, docking studies were performed. These studies give insight about the radical scavenging ability of newly synthesized derivatives **5A** and **5B**. Both of these compounds gave excellent binding affinity for tyrosinase enzyme presenting the energy of -7.325 Kcal/mol and -6.527 Kcal/mol for compounds **5A** and **5B**, respectively, as shown in Figure 5.

Docking poses were analyzed to get the best binding posture and compared with cocrystallized ligand kojic acid [57]. Compound **5A** formed pi-sigma interaction with Val A: 218 having bond length of 3.56 Å, while pi-pi stacked bonding with His A: 208 with bond length of 4.03 Å. Additionally, it formed amide pi-staked linkages with Gly A: 200 and pi-alkyl bindings with Ala A: 221 (5.27 Å bond length) and Pro A: 201 (4.51 Å bond length). Rings of the synthesized chemical moiety provide excellent synergism with the active site resulting in its increased docking results.

Derivative **5B** also gave admirable affinity inside the binding pocket of enzyme making this derivative favorable as compared with standard ligand kojic acid. It formed a conventional hydrogen bond with Asn A: 205 with bond length of 2.44 Å while it formed carbon-hydrogen bond with Met A: 215 (bond length 3.75 Å). Simultaneously, a bonding pattern also consists of alkyl and pi-alkyl linkages with Pro A: 201, Val A: 218, and His A: 208 with bond length ranging from 4.32 Å to 5.19 Å. The results of molecular docking supported the *in vitro* DPPH antioxidant potential.

#### 3.5.3. Molecular Docking Studies of Compound **5A** Using MOE 2016 against AChE, BChE, COX-1, COX-2, and 5-LOX

Compound **5A** was docked into the active sites of AChE, BChE, COX-1, COX-2, and 5-LOX. The three-dimensional structures of all the enzymes were obtained from the Protein Data Bank with accession codes 1EVE, 4BDS, 1EQG, 1CX2, and 6N2W, respectively [58, 59].

**Figure 6:**
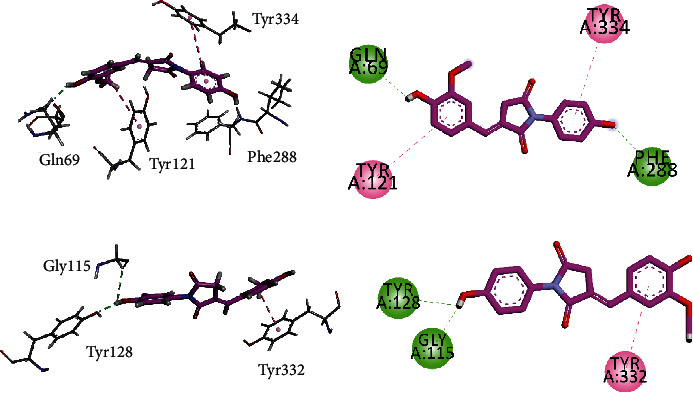


Two/three-dimensional (2-D/3-D) interaction plots of compound **5A** in the binding site of AChE and BChE are shown in Figure 3. Hydroxyl groups of **5A** interact with peripheral anionic site residue (PAS) Gln69 and acyl pocket residue Phe288 via hydrogen bond interaction (Figure 6(a)), while the two phenyl rings interact with PAS residues Tyr121 and Tyr334 *via π*-*π* stacking interaction.  Figure 3(b) shows the interaction plot of **5A** in the binding site of BChE. The hydroxyl group displayed hydrogen bond interactions with Gly115 and Tyr128, while phenyl ring interacts with CAS residue Tyr332 via *π*-*π* stacking interaction (Figure 6(b)).

**Figure 7:**
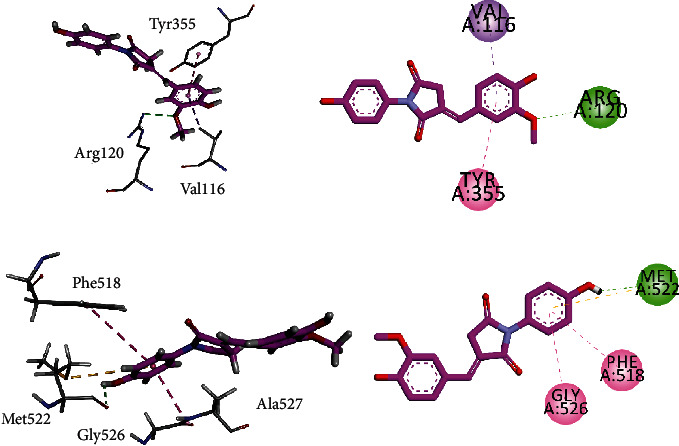


Figures 7(a) and 7(b) show the interaction plots (2-D/3-D) into the binding sites of COX-1 and COX-2. The inhibitor **5A** interacts with the construction site residues Arg120 and Tyr355. Arg120 forms hydrogen bond interactions with methoxy-oxygen atom, while Tyr355 forms *π*-*π* T-shaped interaction with the phenyl ring. 4-Hydroxy-3-methoxyphenyl ring extends to the lobby region and forms *π*-*σ* interaction with Val116 to stabilize the ligand-enzyme complex (Figure 7(a)). The same compound forms *π*-*π* stacked interaction with Gly526 present at the top of the COX-2. Another residue present at the apex of the active site Met522 forms hydrogen bond interactions as well as *π*-sulfur interactions. Methoxyphenyl ring forms contact with the side pocket residue Phe518 (Figure 7(b)).

**Figure 8:**
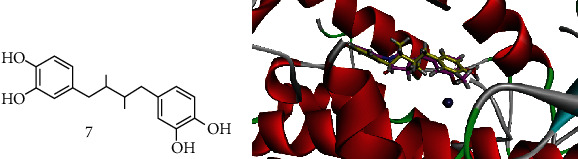


**Figure 9:**
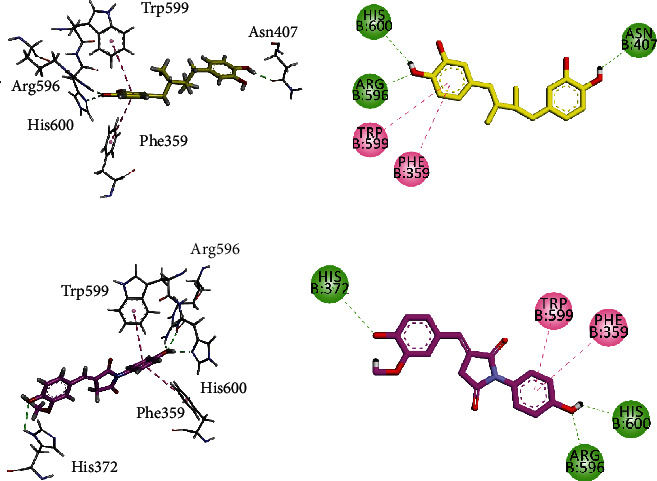


Compound **5A** displayed the highest activity (IC_50_ = 0.71 ± 0.10 *μ*M) against 5-LOX. We selected the 5-LOX enzyme with PDB ID 6N2W as it is cocrystalized with natural product ligand nordihydroguaiaretic acid (NDGA, **7**, Figure 8(a)). The superposed ribbon diagram of compound **5A** and native ligand NDGA is shown in Figures 8 and 9. The diagram showed that compound **5A** occupied the same space with native ligand. 3-D interaction plot revealed that both native and **5A** shared almost the same amino acid residue contact. Both displayed *π*-*π* interactions with Phe359 and Trp599, while Arg596 and His600 formed hydrogen bond interaction with **5A**.

#### 3.5.4. Molecular Docking Studies of Compound **5B** against *α*-Glucosidase, *α*-Amylase, and PTP-1B Using MOE 2016

**Figure 10:**
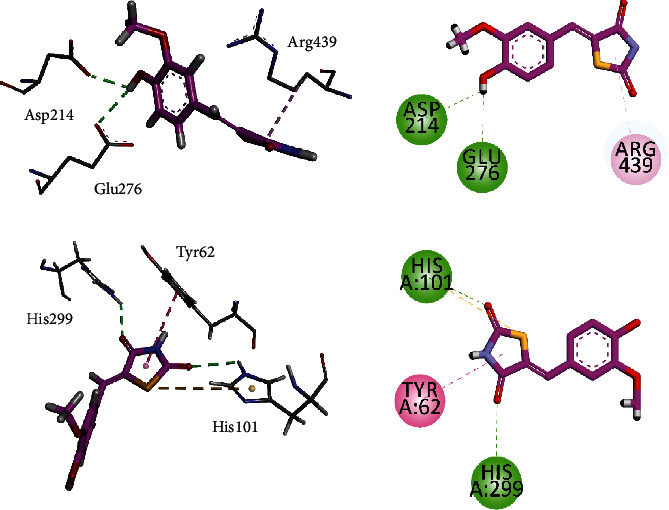


Compound **5B** was docked into the binding sites of *α*-glucosidase, *α*-amylase, and PTP-1B. For this purpose, the homology-modelled structure of *α*-glucosidase previously reported by our research group was used for docking, while 3-D crystal structures of *α*-amylase and PTP-1B were obtained from PDB with accession numbers 4W93 and 1NNY, respectively. The interaction plots of the compounds are shown in Figures 9 and 10. Compound **5B** established contacts with residues present in the catalytic triad of homology-modelled *α*-glucosidase (Figure 9(a)). The hydroxyl group forms hydrogen bond interactions with Asp214 and Glu276, while Arg439 forms *π*-alkyl interactions with thiazolidine ring. For *α*-amylase, compound **5B** forms two hydrogen bond interactions with His101 and His299, while Tyr62 forms *π*-*π* interactions to stabilize the ligand-enzyme complex (Figure 9(b)).

**Figure 11:**
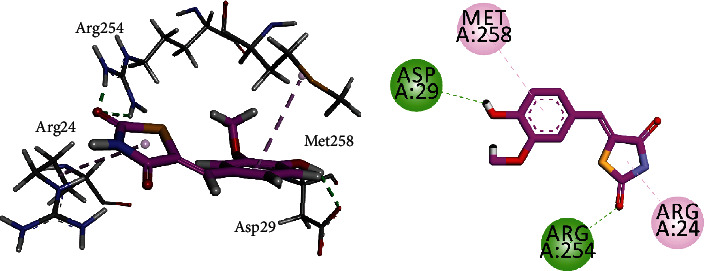


Compound **5B** was docked into the binding site of protein tyrosine phosphatase 1B (PTP1B). The 3-D/2-D interaction plots of the compound into the binding site of 1NNY are shown in Figure 11. Compound **5A** showed two hydrogen bond interactions with Asp29 and Arg254, while Arg24 and Met258 form *π*-alkyl interactions.

## 4. Conclusions

In conclusion, we have isolated six antioxidant compounds (**1–6**) from *Polygonum aviculare*. Two of the isolated compounds (**1**-**2**) are alcohol analogs of furan, and four (**3–6**) were phenols. The most active antioxidant compound **5** (IC_50_ = 2.12 *μ*g/mL) was selected for further derivatization. Based on the important pharmacophoric features of a number of drugs containing succinimide and thiazolidine, two derivatives **5A** and **5B** were designed and synthesized. These rationally designed derivatives were emerged as good to excellent inhibitors of all the tested targets. The hydroxy succinimide derivative **5A** was found to be a potent inhibitor of AChE, BChE, COX-1, COX-2, 5-LOX, and DPPH, while thiazolidine analog **5B** was found to be a potent multitarget antidiabetic agent. Docking studies were also performed against all the tested targets to explore the binding orientation/affinity and possible mechanism of inhibition. All the tested compounds were observed to have excellent binding affinity towards protein receptors.

## Figures and Tables

**Table 1:** Percent ABTS and DPPH inhibition activities of isolated compounds (**1–6**).

Comp No.	Structure	ABTS IC_50_*μ*g/ml	DPPH IC_50_*μ*g/ml
1	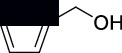	7.43	8.19
2	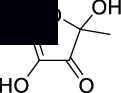	3.005	1.61
3	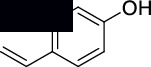	12.60	10.27
4	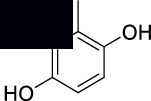	4.63	3.19
5	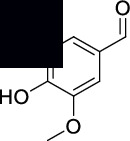	8.28	2.12
6	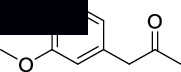	9.55	9.99
Ascorbic acid		0.211	0.840

**Table 2:** Enzymatic activities of compound **5A**.

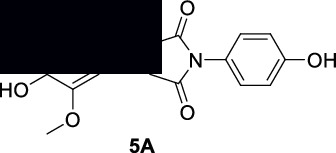								
Sample	AChE IC_50_	BChE IC_50_	SI	COX-1 IC_50_	COX-2 IC_50_	SI	5-LOX IC_50_	DPPH IC_50_
5A	10.60 ± 1.03	15.10 ± 1.21	1.42	13.91 ± 1.67	1.08 ± 0.05	12.9	0.71 ± 0.10	1.05 ± 0.51
Galantamine	4.0 ± 0.10	15.0 ± 0.67	3.75	—	—	—	—	—
Diclofenac				4.48 ± 0.10	10.80 ± 0.71	2.4	—	
Zileuton	—	—	—	—	—	—	5.29 ± 0.19	—
Ascorbic acid	—	—	—	—	—	—	—	0.840 ± 0.14

**Table 3:** In vitro antidiabetic activities of compound **5B**.

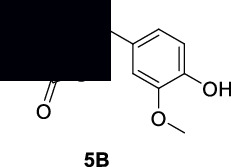				
Sample	*α*-Glucosidase IC_50_	*α*-Amylase IC_50_	PTP1B IC_50_	DPPH IC_50_
5B	15.34 ± 1.08	21.83 ± 1.10	53.28 ± 1.16	1.94 ± 1.20
Acarbose	10.60 ± 0.21	12.71 ± 0.08	—	—
Ursolic acid	—	—	3.50 ± 0.23	—
Ascorbic acid	—	—	—	0.840 ± 0.04

**Table 4:** Binding energies, score, and interaction types of synthesized compounds (**1-6**) with amino acid residues at active site.

Compound	Binding energy (Kcal/mol)	Score	Types of interactions	Amino acid residues
1	-5.264	-49.392	Hydrogen bonds, polar bonds, Van der Waal forces, *π*-*π* interactions	His A: 42, His A: 60, Val A: 204, Val A: 208, Asn A: 205, Arg A: 209
2	-6.987	-33.427	*π*-*π* interactions, carbon hydrogen bond, alkyl bond	His A: 60, His A: 204, Val A: 218
3	-5.368	-37.856	*π*-Alkyl bond, conventional hydrogen bond, Van der Waal forces	His A: 42, His A: 60, Val A: 204, Val A: 218, Phe A: 227
4	-7.124	-39.834	Conventional hydrogen bond, carbon-hydrogen bond, *π*-alkyl bond, Van der Waal forces	His A: 60, His A: 204, His A: 208, Met A: 215, Val A: 218
5	-5.427	-41.347	*π*-Alkyl bond, conventional hydrogen bond, Van der Waal forces	His A: 42, Val A: 204, His A: 205, Asn A: 205, Phe A: 227
6	-4.125	-37.785	Conventional hydrogen bond, carbon-hydrogen bond, *π*-alkyl bond, *π*-*π* interactions	His A: 42, His A: 204, Ala A: 221, Phe A: 227, Arg A: 209

## Data Availability

The whole data is available in the manuscript and supporting file.

## Supplementary Materials

Supplementary Materials MS, ^1^H, and ^13^C NMR spectra of the compounds. Supplemental files: Figure S1: MS chromatogram of compound **1**. Figure S2: ^1^H NMR spectrum of compound **1**. Figure S3: ^13^C NMR spectrum of compound **1**. Figure S4: MS chromatogram of compound **2**. Figure S5: ^1^H NMR spectrum of compound **2**. Figure S6: ^13^C NMR spectrum of compound **2**. Figure S7: MS chromatogram of compound **3**. Figure S8: ^1^H NMR spectrum of compound **3**. Figure S9: ^13^C NMR spectrum of compound **3**. Figure S10: MS chromatogram of compound **4**. Figure S11: ^1^H NMR spectrum of compound **4**. Figure S12: ^13^C NMR spectrum of compound **4**. Figure S13: MS chromatogram of compound **5**. Figure S14: ^1^H NMR spectrum of compound **5**. Figure S15: ^13^C NMR spectrum of compound **5**. Figure S16: MS chromatogram of compound **6**. Figure S17: ^1^H NMR spectrum of compound **6**. Figure S18: ^13^C NMR spectrum of compound **6**. Figure S19: ^1^H NMR spectrum of compound **5A**. Figure S20: ^13^C NMR spectrum of compound **5A**. Figure S21: ^1^H NMR spectrum of compound **5B**. Figure S22: ^13^C NMR spectrum of compound **5B**.
