# Supplementary material for: Antioxidant Molecules Isolated from Edible Prostrate Knotweed: Rational Derivatization to Produce More Potent Molecules
Source: Oxid Med Cell Longev. 2022 Feb 27;2022:3127480. doi: 10.1155/2022/3127480 (PMC9020998; doi:10.1155/2022/3127480)
Supplement: Supplementary Materials — MS, 1H, and 13C NMR spectra of the compounds. Supplemental files: Figure S1: MS chromatogram of compound 1. Figure S2: 1H NMR spectrum of compound 1. Figure S3: 13C NMR spectrum of compound 1. Figure S4: MS chromatogram of compound 2. Figure S5: 1H NMR spectrum of compound 2. Figure S6: 13C NMR spectrum of compound 2. Figure S7: MS chromatogram of compound 3. Figure S8: 1H NMR spectrum of compound 3. Figure S9: 13C NMR spectrum of compound 3. Figure S10: MS chromatogram of compound 4. Figure S11: 1H NMR spectrum of compound 4. Figure S12: 13C NMR spectrum of compound 4. Figure S13: MS chromatogram of compound 5. Figure S14: 1H NMR spectrum of compound 5. Figure S15: 13C NMR spectrum of compound 5. Figure S16: MS chromatogram of compound 6. Figure S17: 1H NMR spectrum of compound 6. Figure S18: 13C NMR spectrum of compound 6. Figure S19: 1H NMR spectrum of compound 5A. Figure S20: 13C NMR spectrum of compound 5A. Figure S21: 1H NMR spectrum of compound 5B. Figure S22: 13C NMR spectrum of compound 5B. [file 3127480.f1.docx]

**Antioxidant molecules isolated from edible prostrate knotweed: Rational derivatization to produce more potent molecules**

Mater H. Mahnashi^1^, Bandar A. Alyami^1^, Yahya S. Alqahtani^1^, Ali O. Alqarni^1^, Muhammad Saeed Jan^2^, Fida Hussain^2^, Rehman Zafar^3^, Umer Rashid^4^, Muhammad Abbas^5^, Muhammad Tariq*^6^ and Abdul Sadiq*^7^

^1^Department of Pharmaceutical Chemistry, College of Pharmacy, Najran University, Najran, Saudi Arabia. ^2^Department of Pharmacy, University of Swabi, KP, Pakistan. ^3^Department of Pharmaceutical Chemistry, Faculty of Pharmaceutical Sciences, Riphah International University, Islamabad 44000 Pakistan. ^4^Department of Chemistry, COMSATS University Islamabad, Abbottabad Campus, 22060 Abbottabad, Pakistan. ^5^Department of Pharmacy, Abdul Wali Khan University Mardan, 23200 Mardan, KP, Pakistan. ^6^Department PCB, Rokhan University, Jalalabad, Nangrahar, Afghanistan. ^7^Department of Pharmacy, Faculty of Biological Sciences, University of Malakand, Chakdara, 18000 Dir (L), KP, Pakistan.

**Corresponding authors:** *Muhammad Tariq, Department of PCB, Rokhan University, Jalalabad, Nangrahar, Afghanistan; *Dr. Abdul Sadiq, Associate Professor, Department of Pharmacy, University of Malakand, Chakdara, 18000 Dir (L), KP, Pakistan, Contact: +92(0)301 2297 102, Email: [sadiquom@yahoo.com](mailto:sadiquom@yahoo.com) Dr. Umer Rashid, Assistant Professor, COMSATS University Islamabad, Abbottabad Campus, Pakistan.

**Supporting Information**


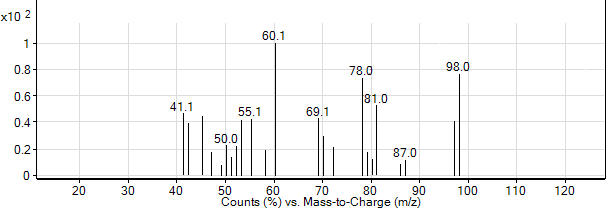


**Figure S1:** MS chromatogram of compound **1**.


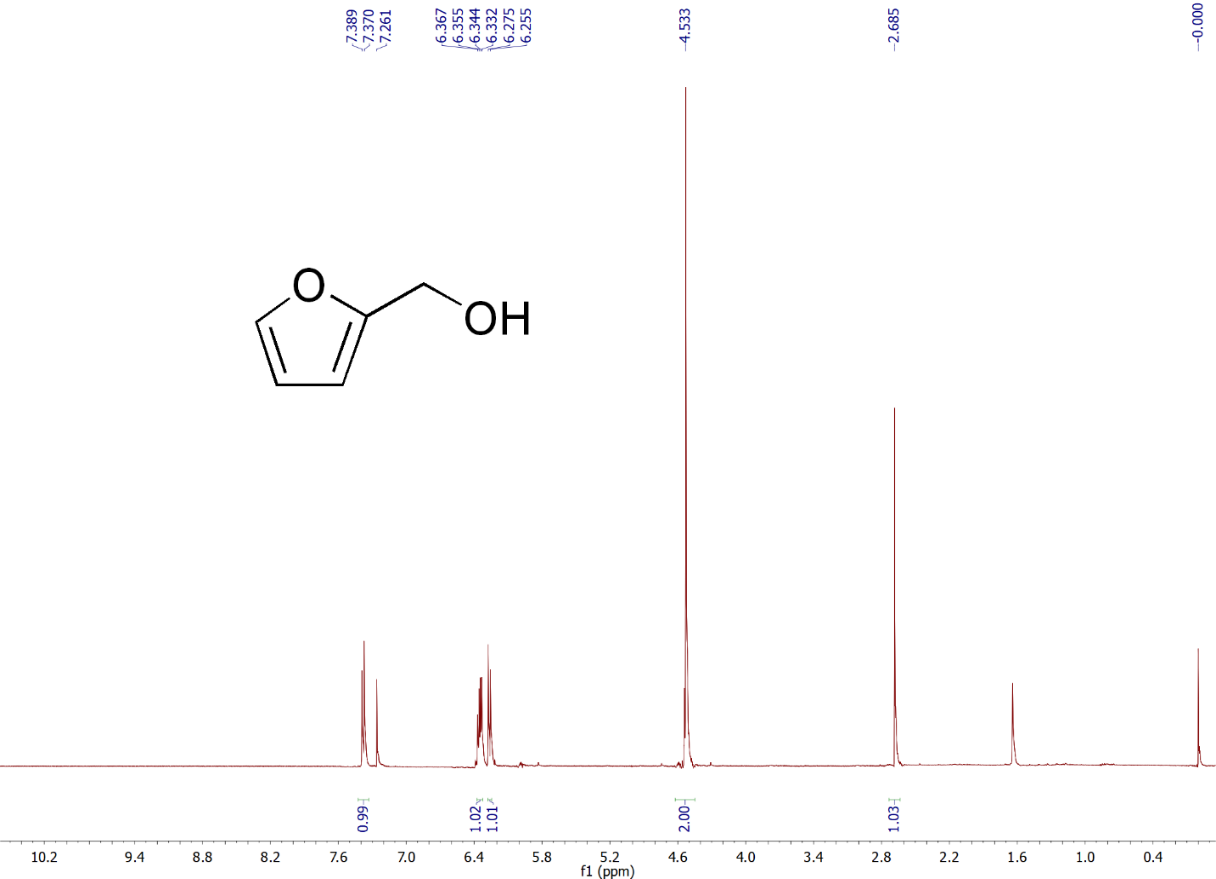


**Figure S2:** ^1^H NMR spectrum of compound **1**.


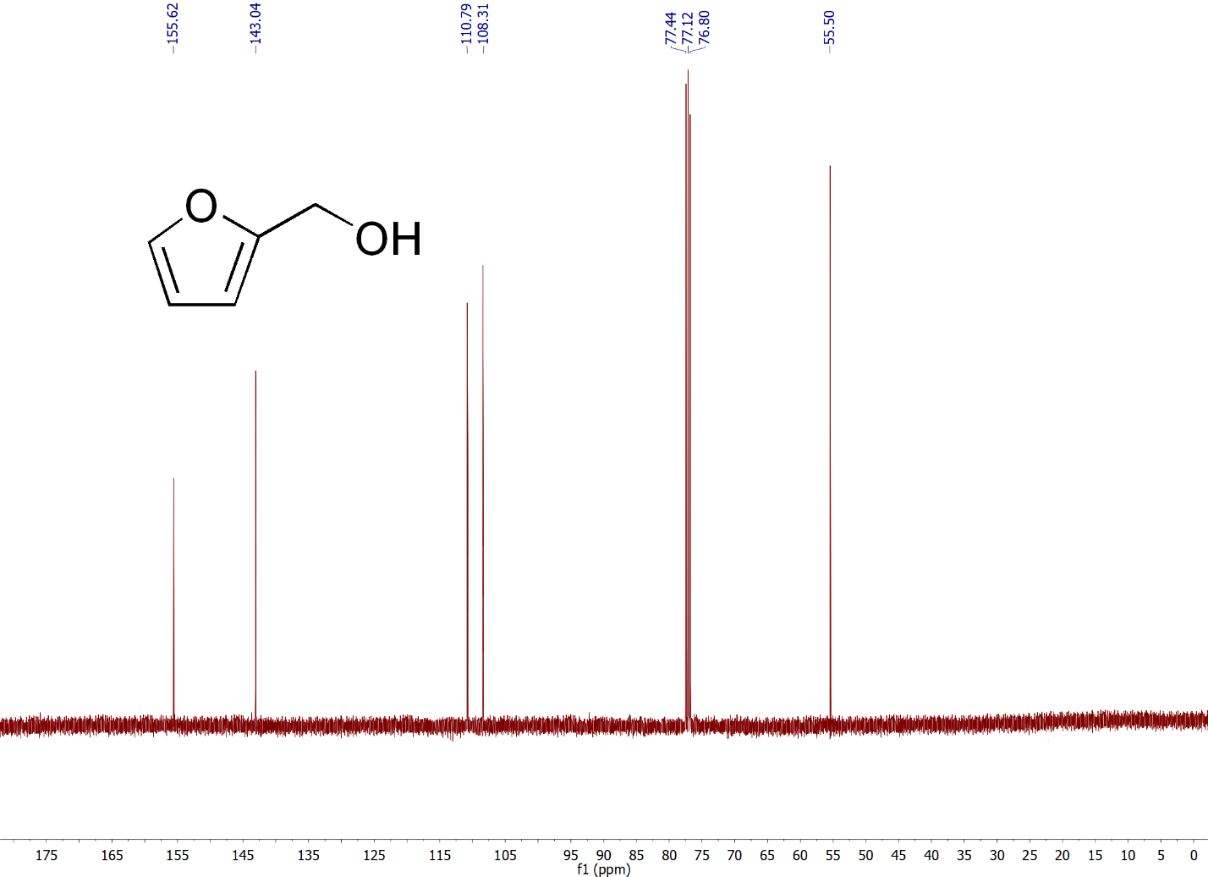


**Figure S3:** ^13^C NMR spectrum of compound **1**.


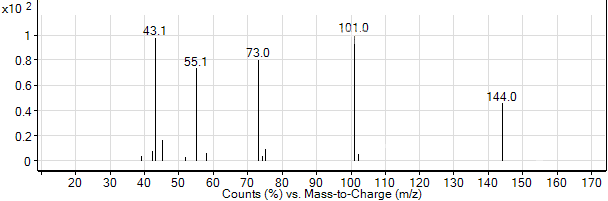


**Figure S4:** MS chromatogram of compound **2**.


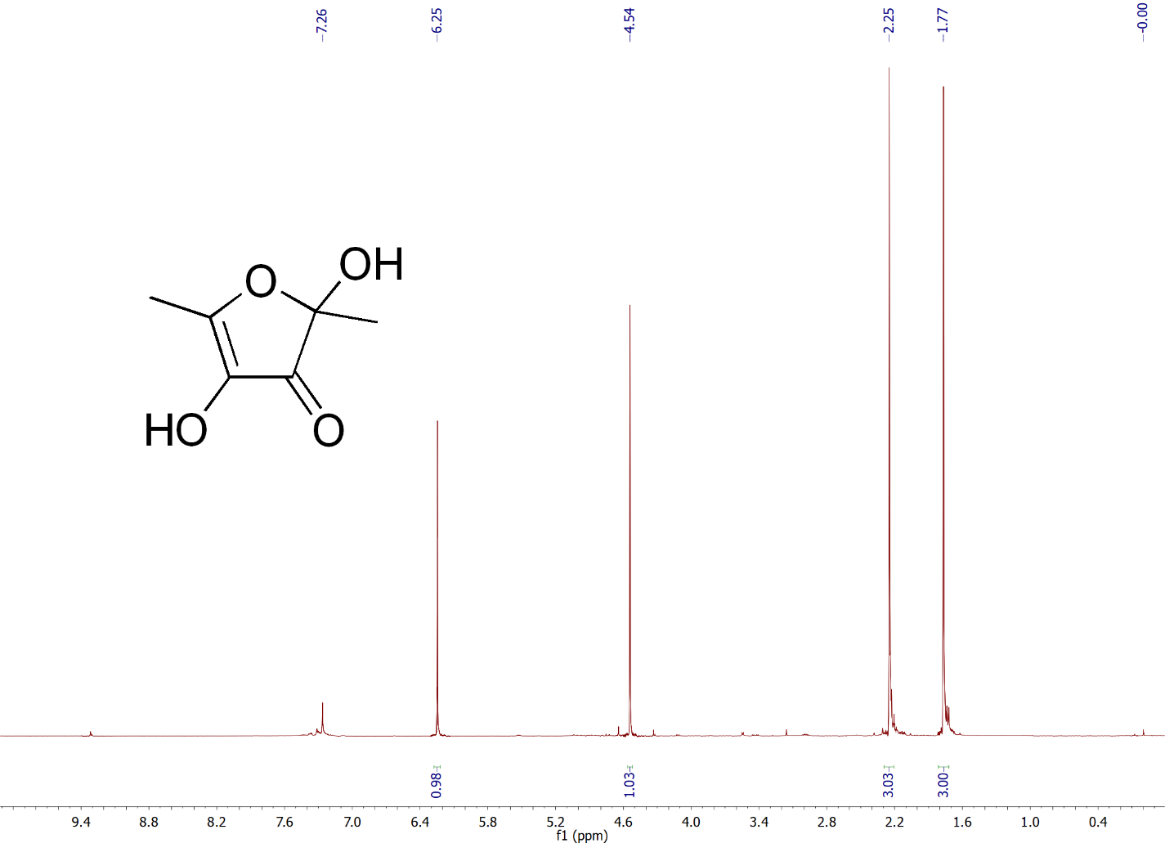


**Figure S5:** ^1^H NMR spectrum of compound **2**.


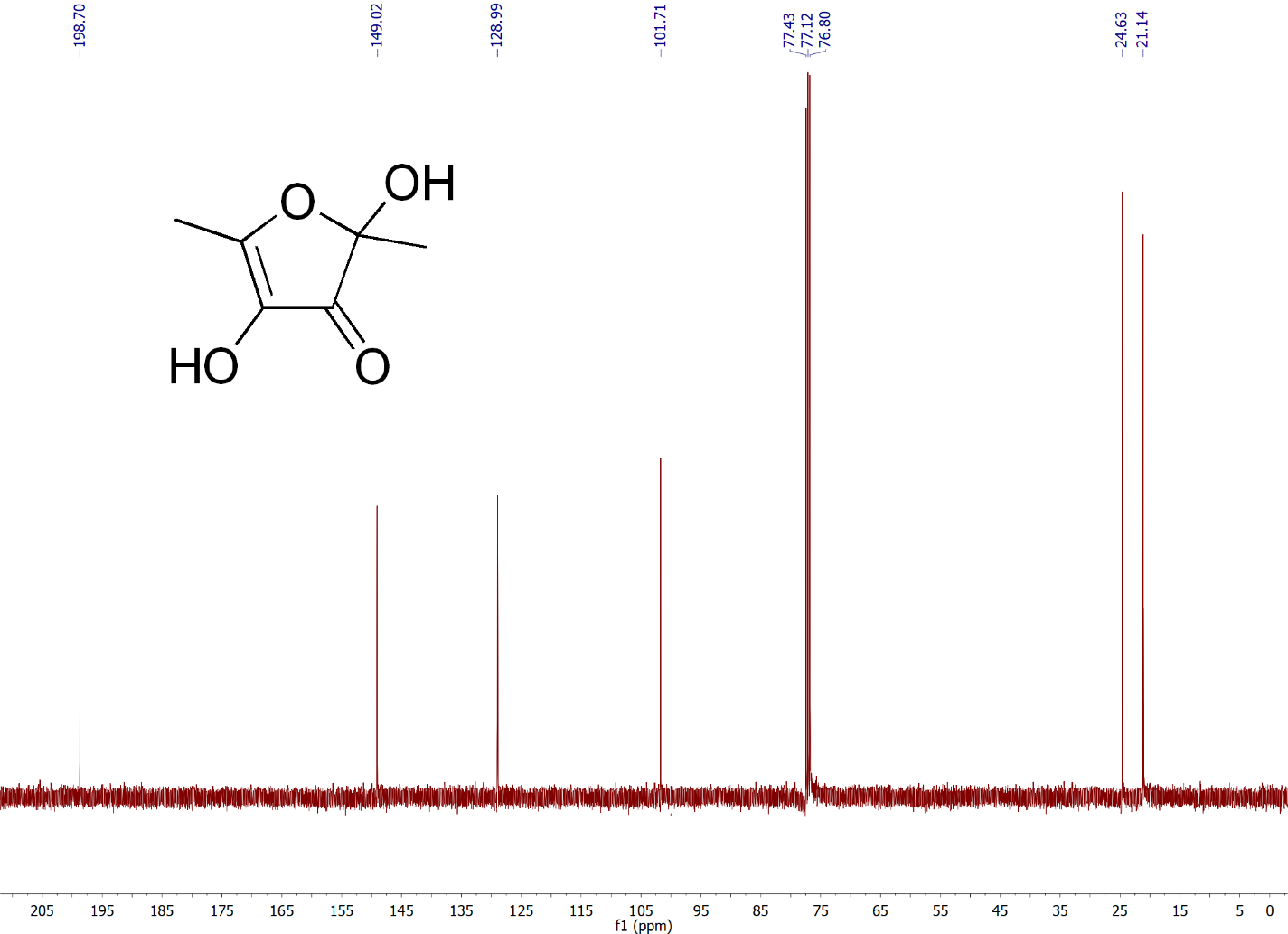


**Figure S6:** ^13^C NMR spectrum of compound **2**.


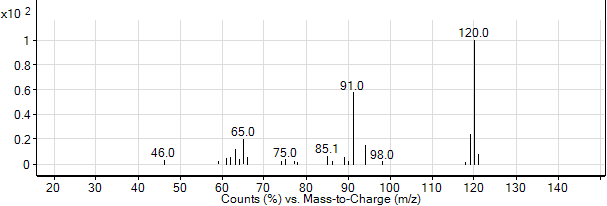


**Figure S7:** MS chromatogram of compound **3**.


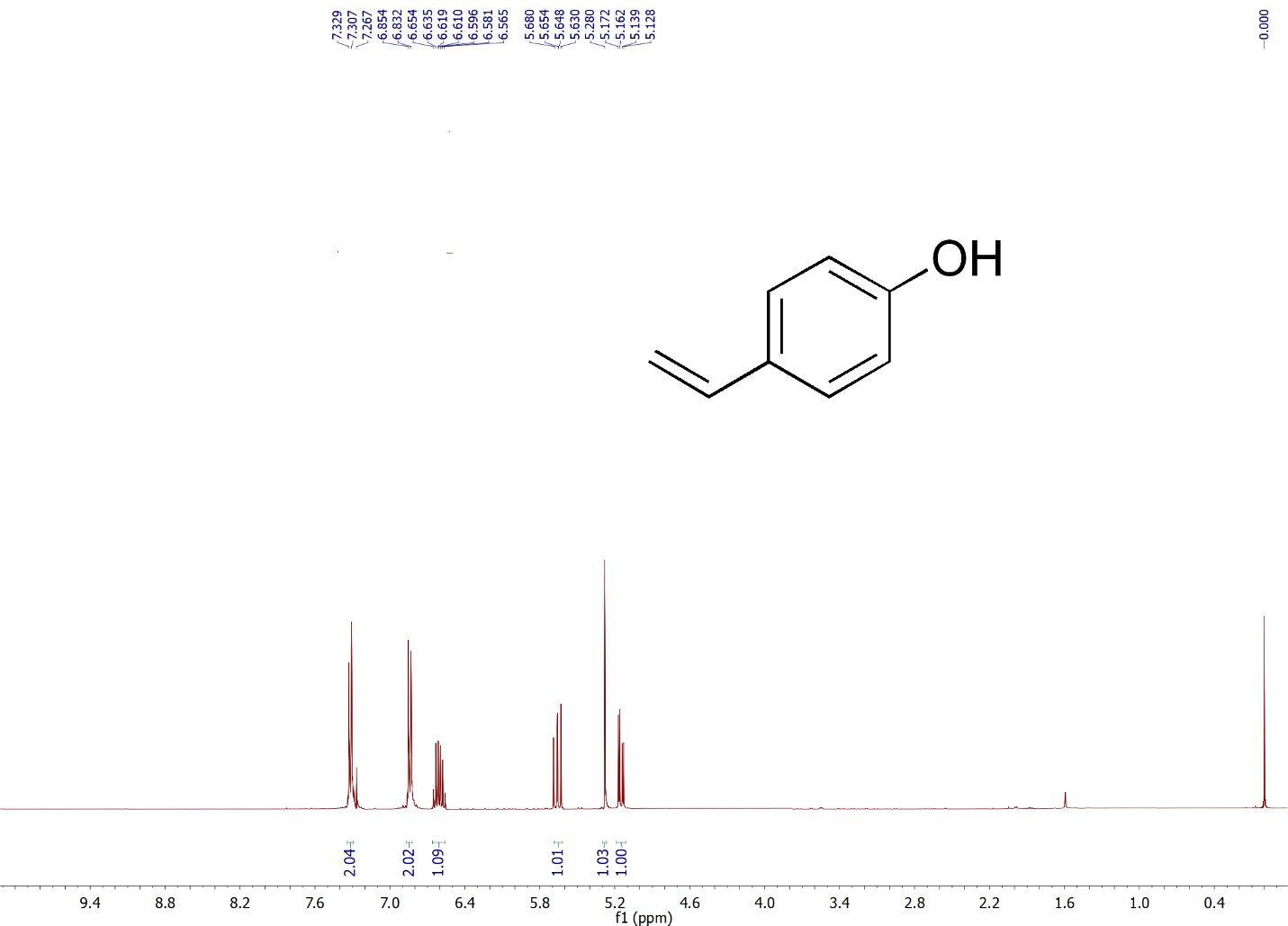


**Figure S8:** ^1^H NMR spectrum of compound **3**.


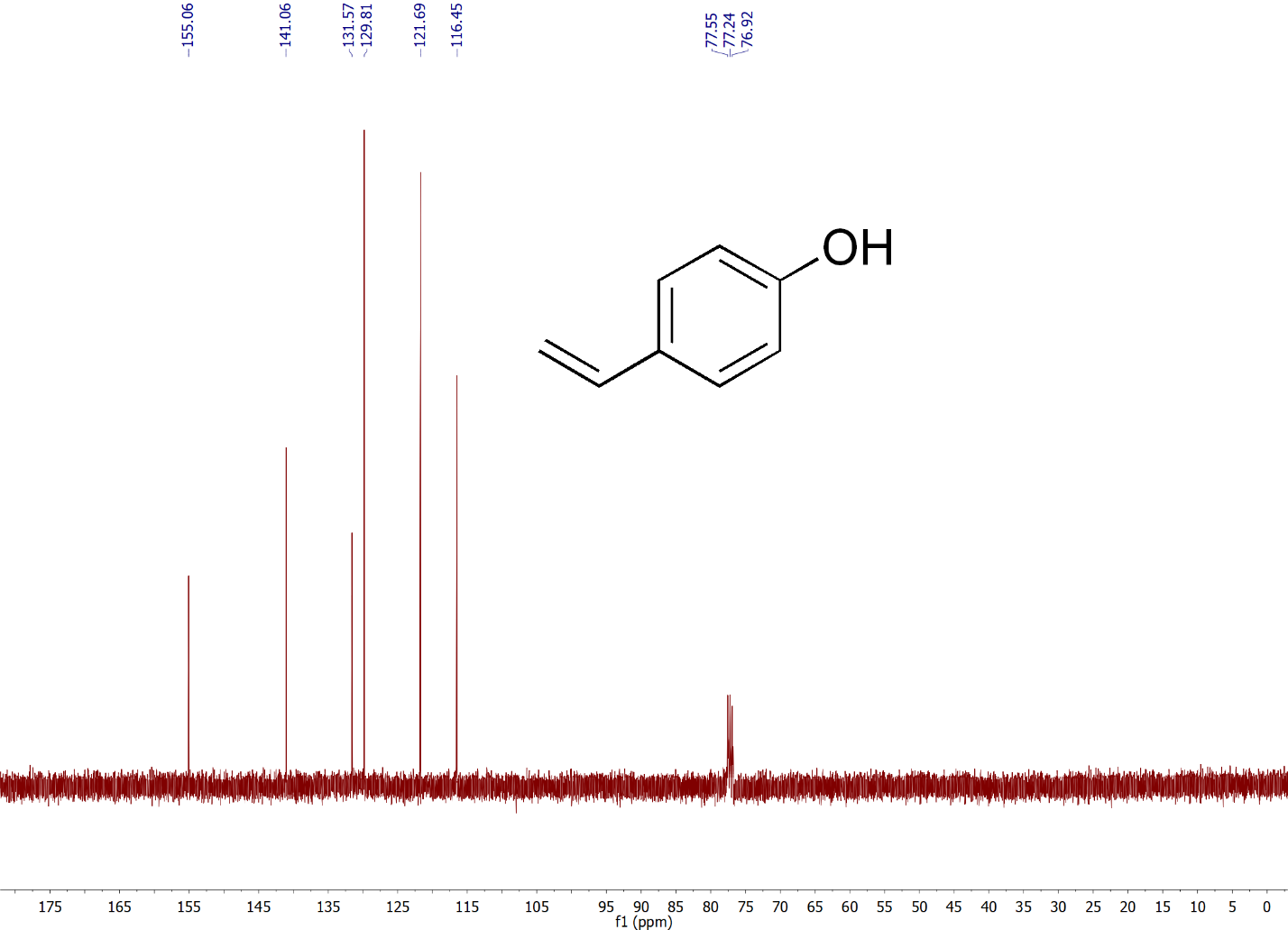


**Figure S9:** ^13^C NMR spectrum of compound **3**.


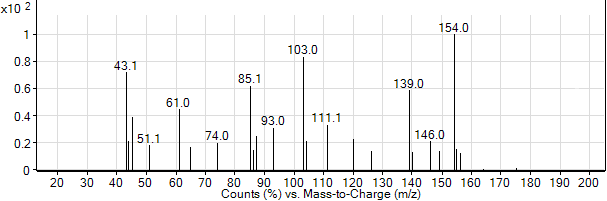


**Figure S10:** MS chromatogram of compound **4**.


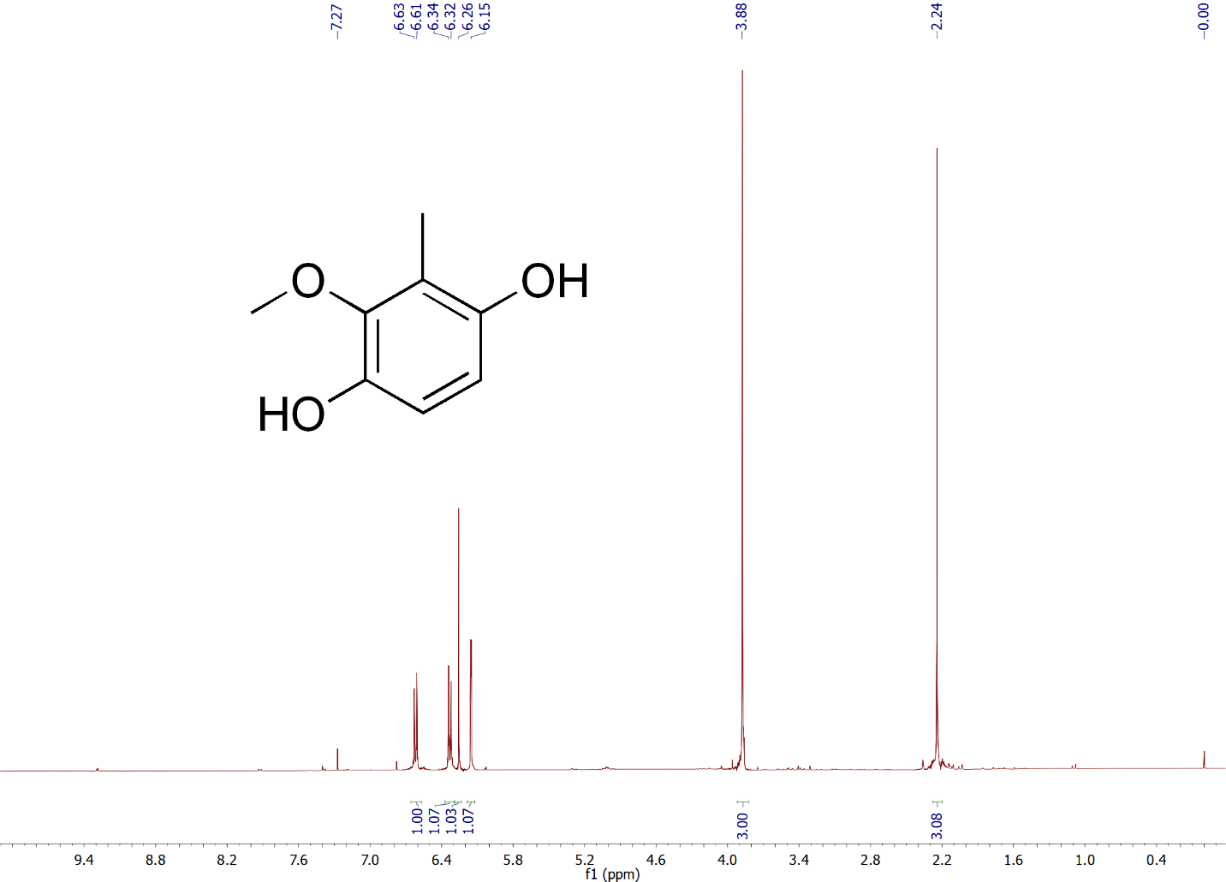


**Figure S11:** ^1^H NMR spectrum of compound **4**.


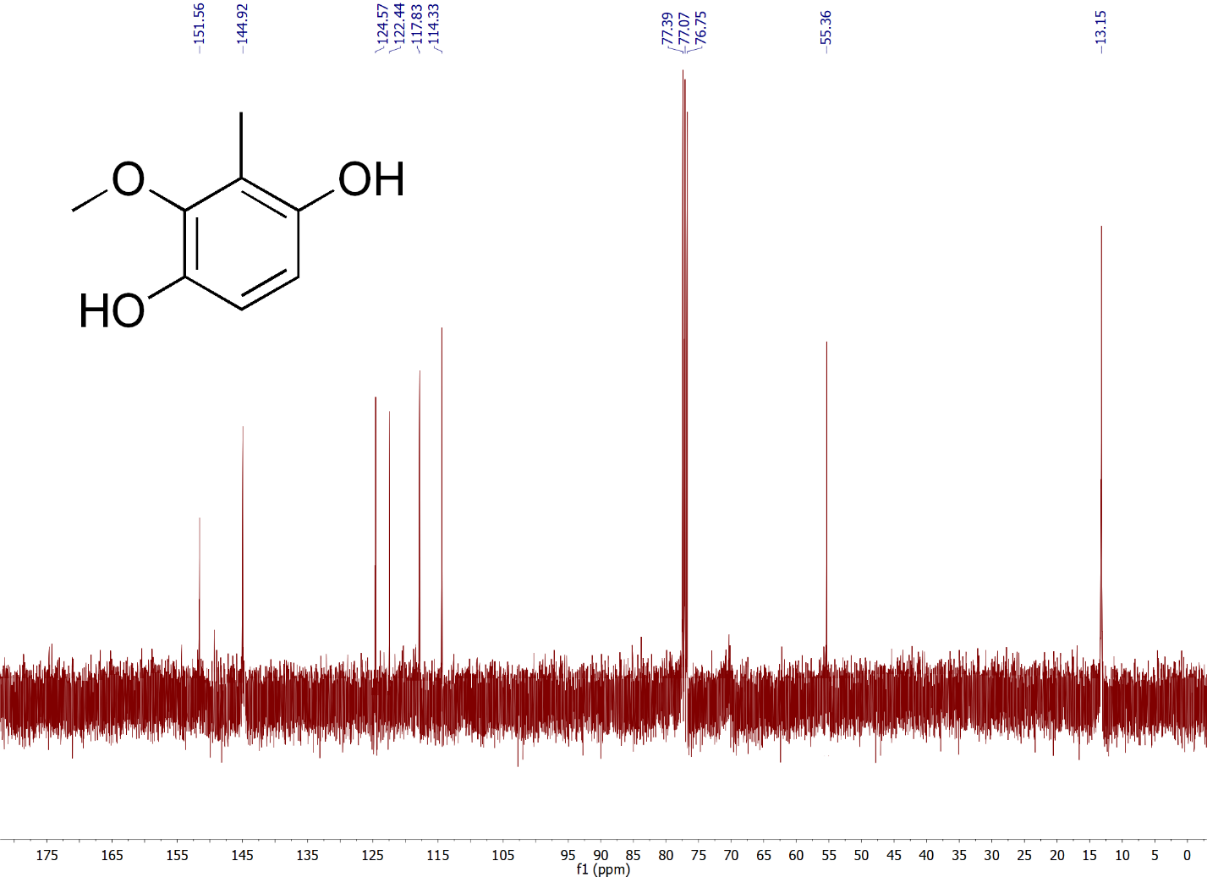


**Figure S12:** ^13^C NMR spectrum of compound **4**.


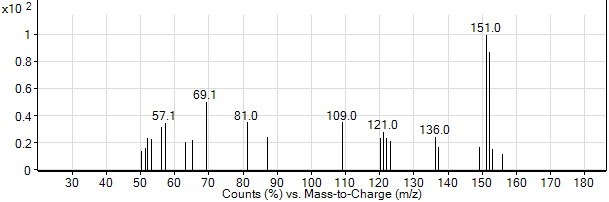


**Figure S13:** MS chromatogram of compound **5**.


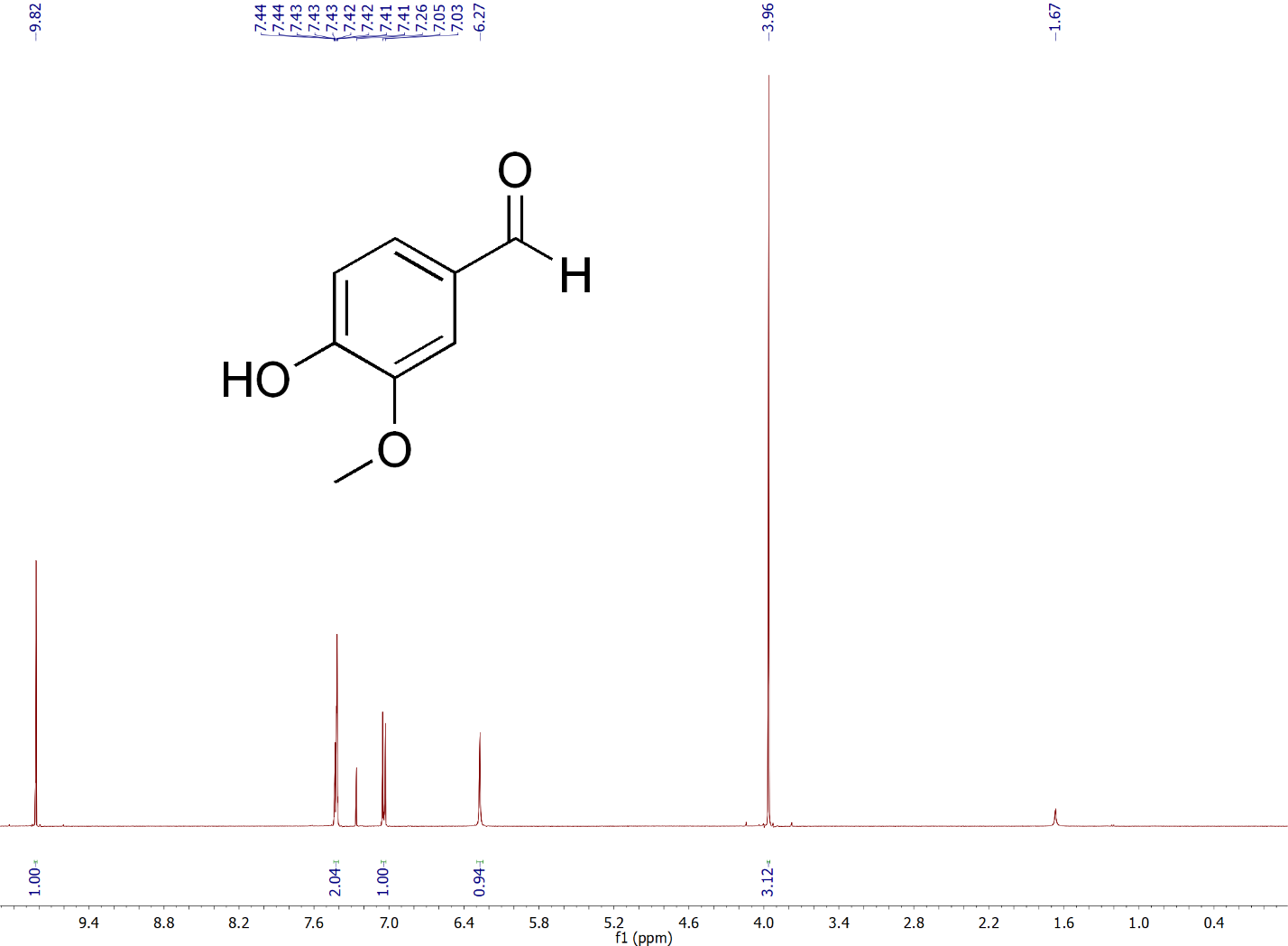


**Figure S14:** ^1^H NMR spectrum of compound **5**.


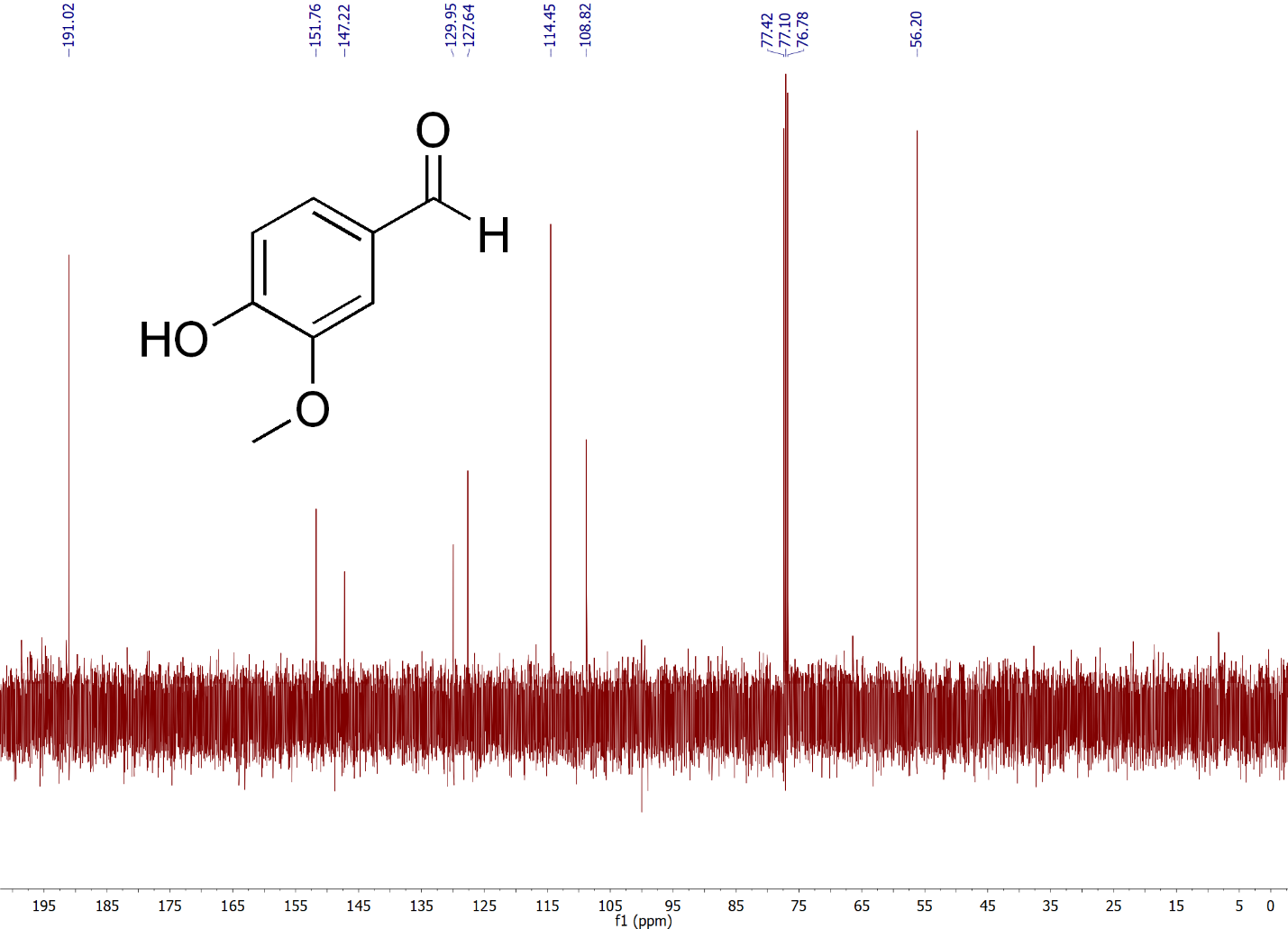


**Figure S15:** ^13^C NMR spectrum of compound **5**.


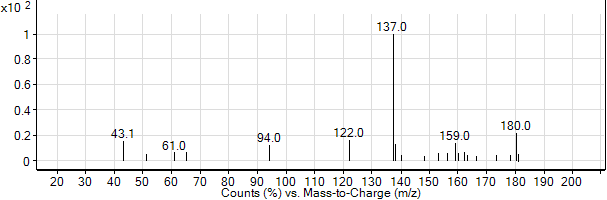


**Figure S16:** MS chromatogram of compound **6**.


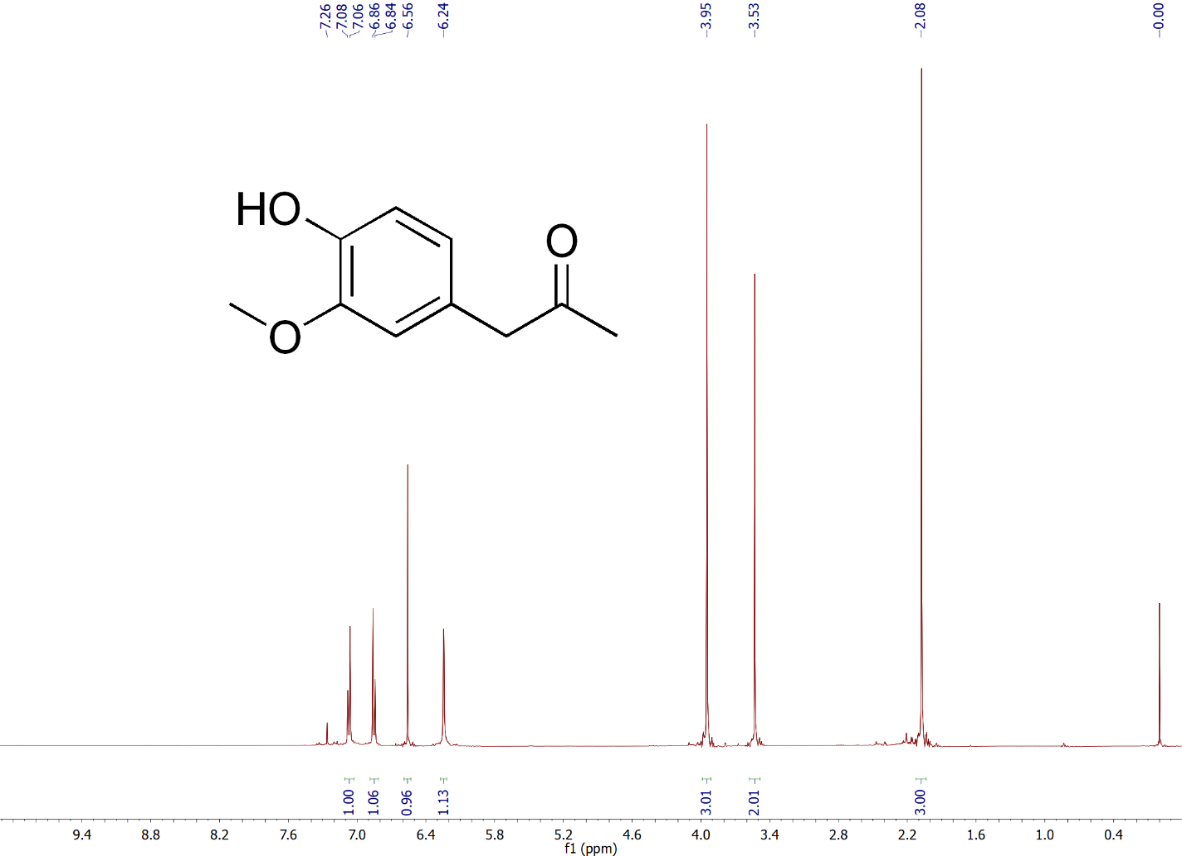


**Figure S17:** ^1^H NMR spectrum of compound **6**.


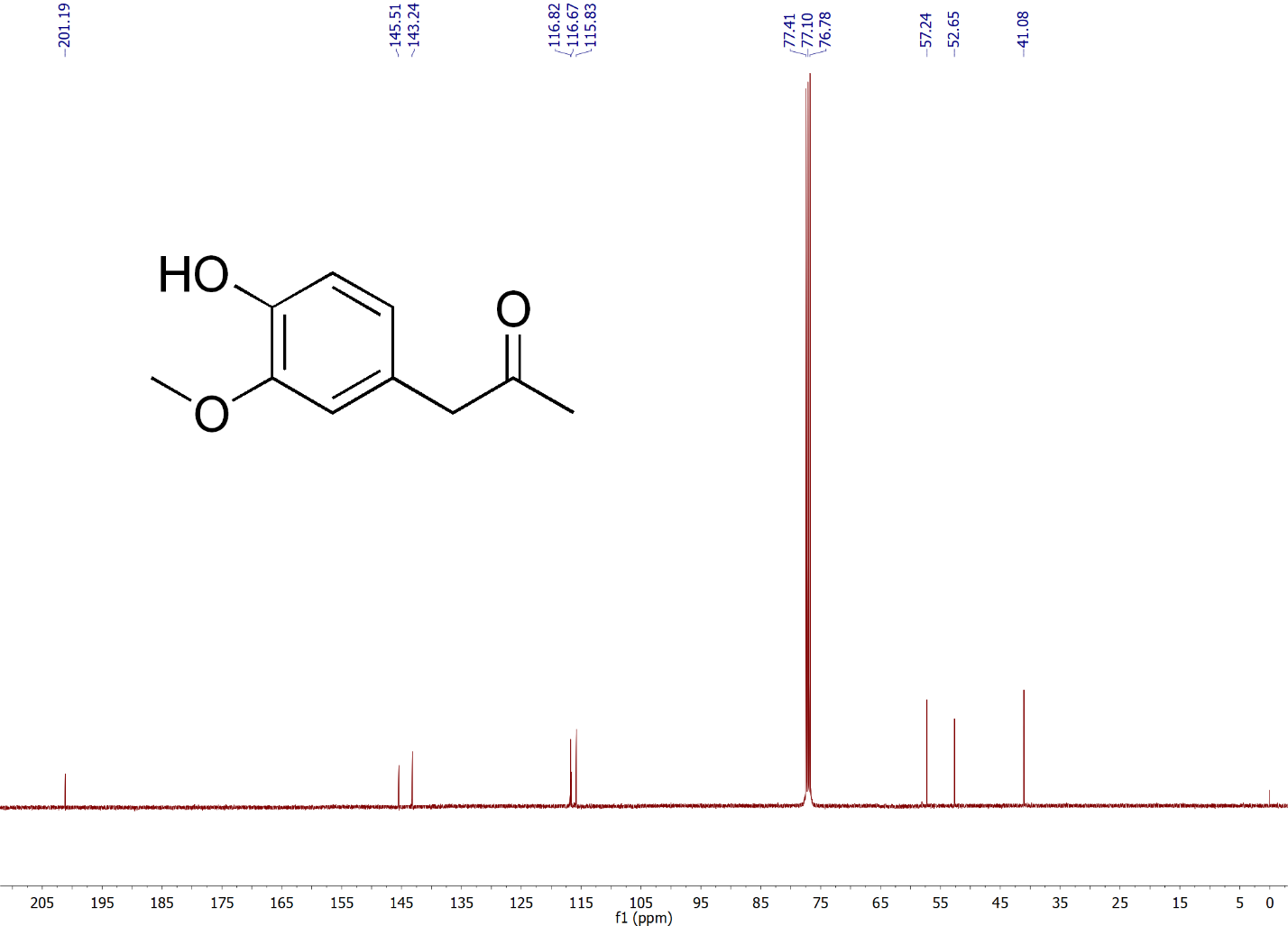


**Figure S18:** ^13^C NMR spectrum of compound **6**.


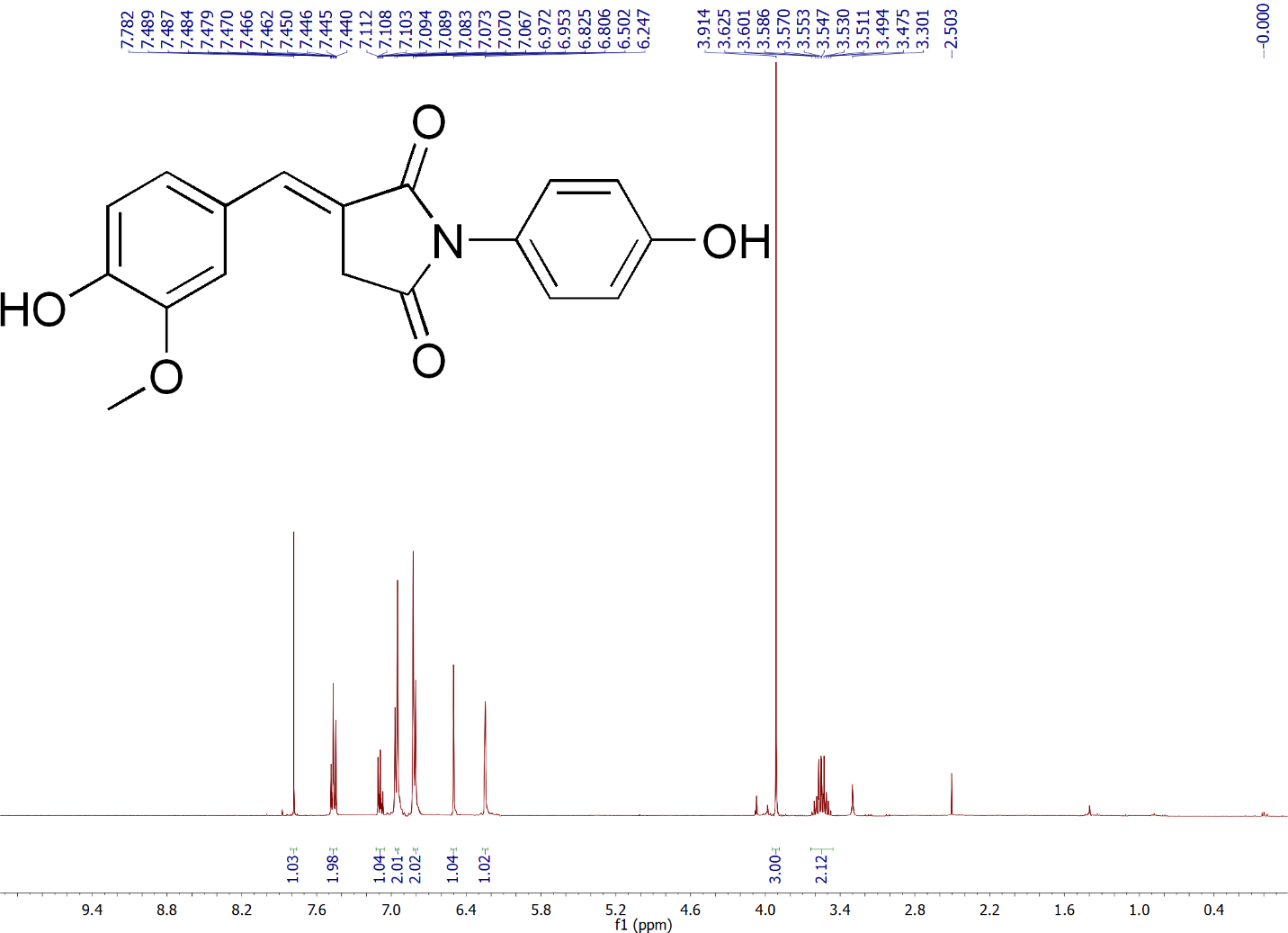


**Figure S19:** ^1^H NMR spectrum of compound **5A**.


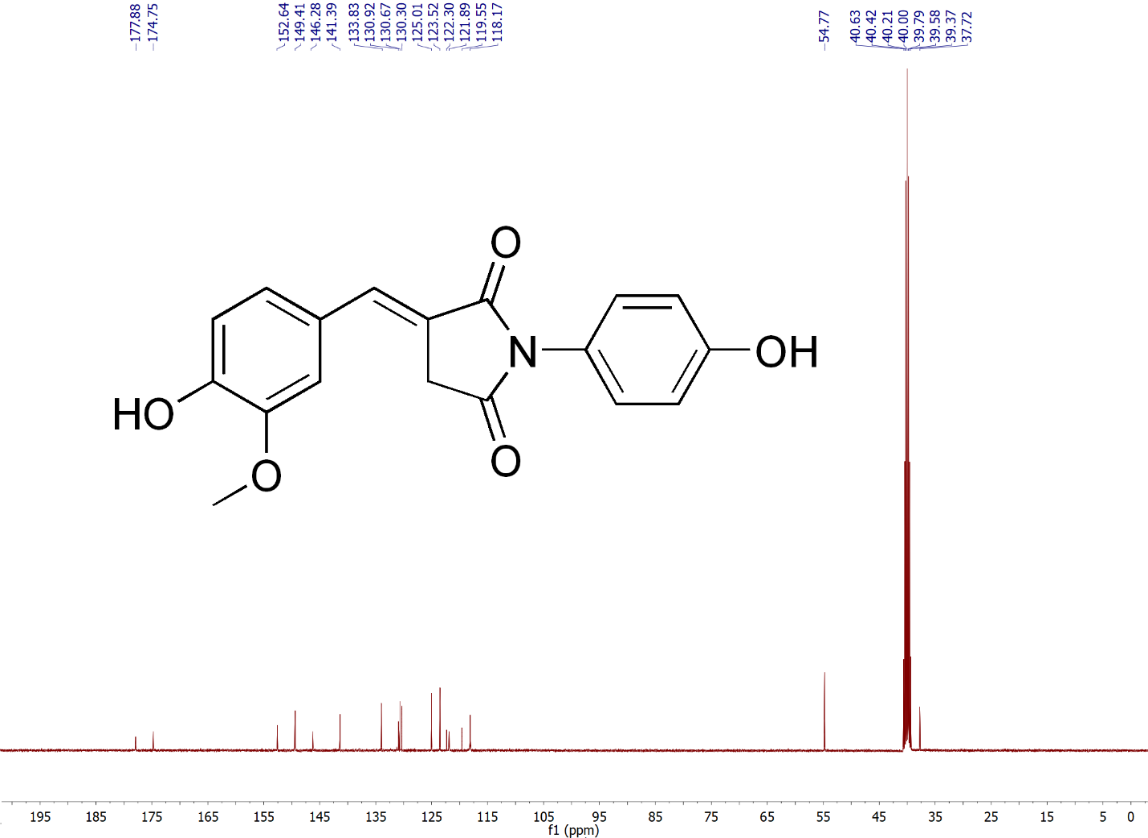


**Figure S20:** ^13^C NMR spectrum of compound **5A**.


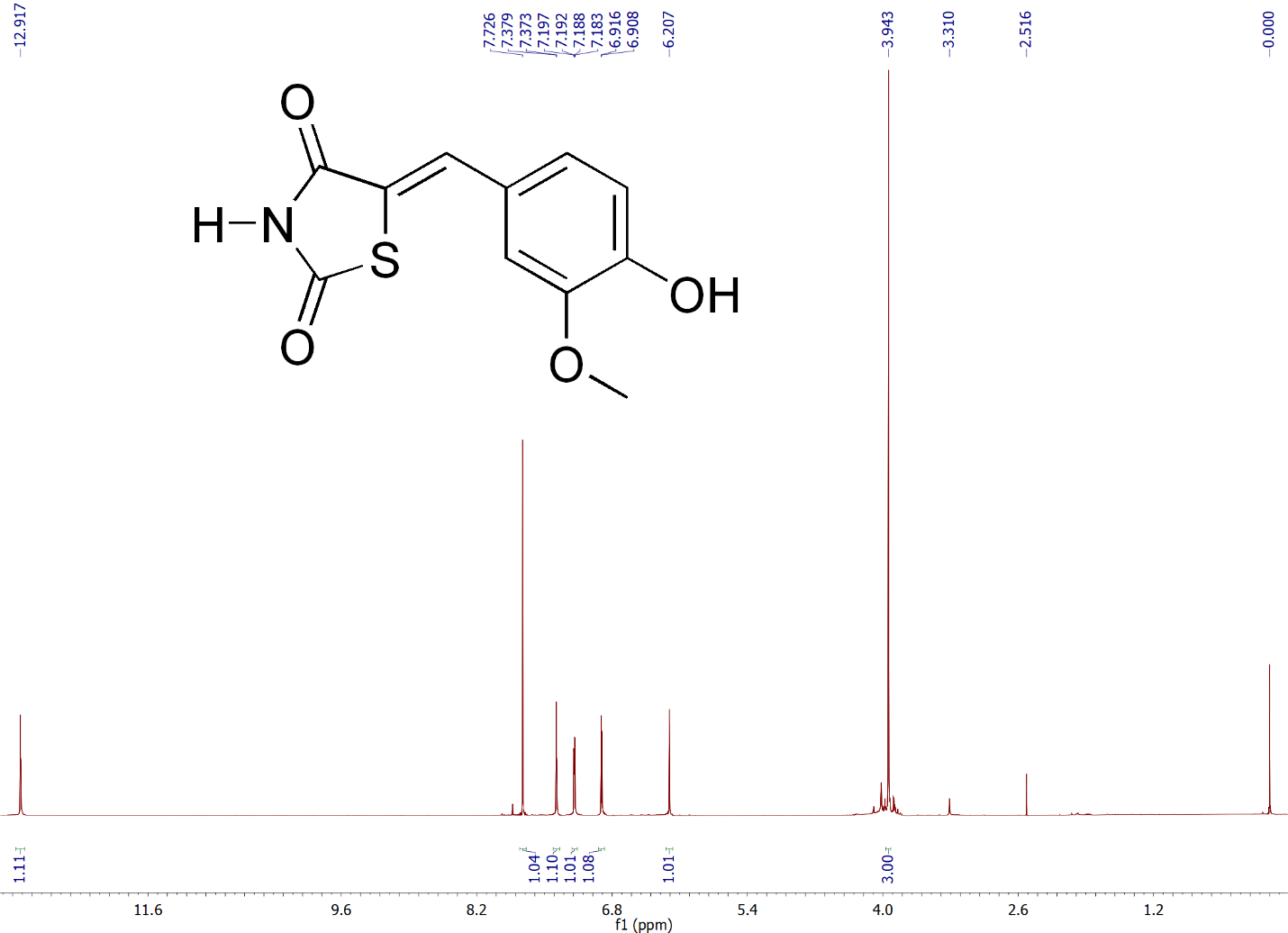


**Figure S21:** ^1^H NMR spectrum of compound **5B**.


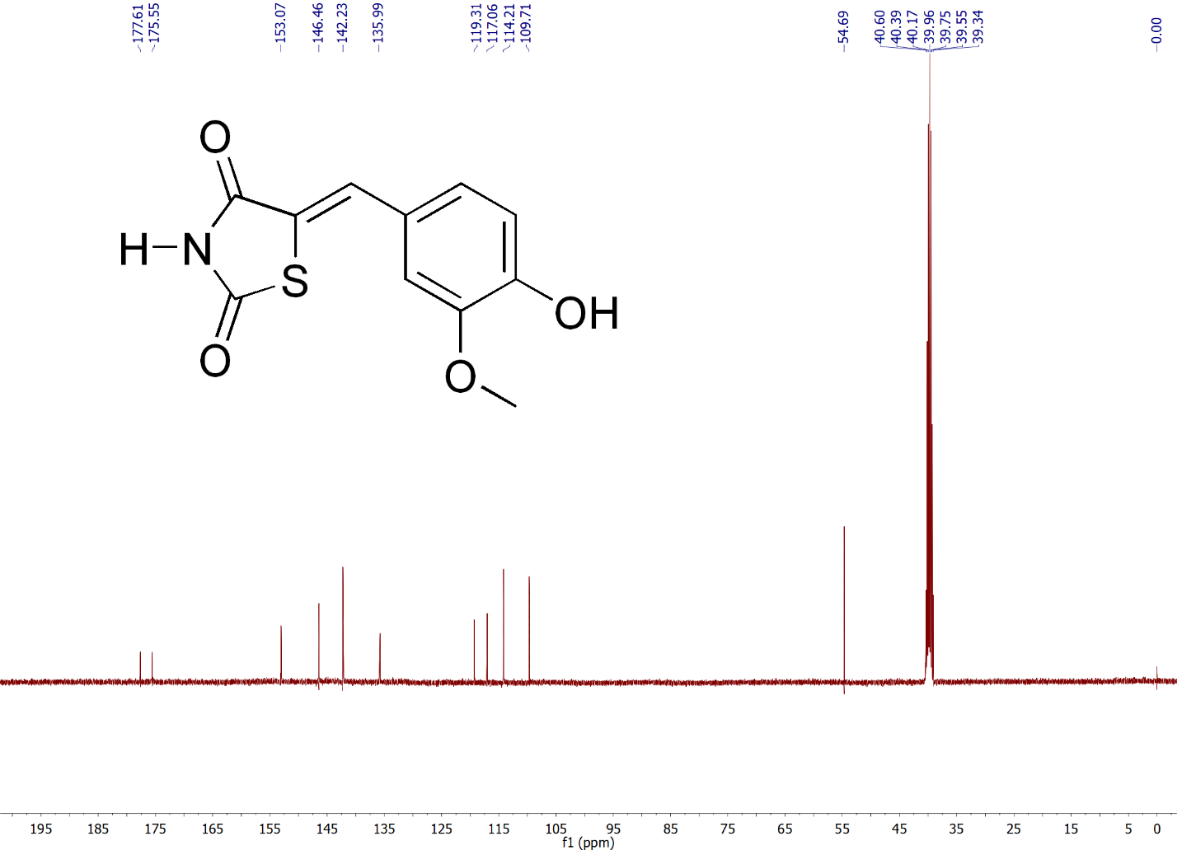


**Figure S22:** ^13^C NMR spectrum of compound **5B**.
